# PANDIA: Personalized neuro-symbolic multimodal fusion for interpretable neonatal pain assessment

**DOI:** 10.1371/journal.pdig.0001442

**Published:** 2026-05-26

**Authors:** Oussama El Othmani, Sami Naouali

**Affiliations:** 1 Computer Science Department, Military Academy of Fondouk Jedid, Nabeul, Tunisia; 2 Military Research Center, Aouina, Tunisia; 3 Information Systems Department, College of Computer Science and Information Technology, King Faisal University, Al Ahsa, Saudi Arabia; Zhujiang Hospital of Southern Medical University, CHINA

## Abstract

Effective pain assessment in infants aged 0–3 months is a critical challenge in neonatal intensive care units (NICUs) and family medicine clinics, where self-reporting is impossible and current observational tools remain subjective and inconsistent. This paper presents PANDIA (Personalized Adaptive Neuro-symbolic Data-fusion for Infant Assessment), a novel multimodal AI system that combines hierarchical representation learning, graph-based inter-modal reasoning, meta-learning personalization, and symbolic concept-bottleneck explanations for robust infant pain assessment. Unlike transformer-centric approaches, PANDIA employs lightweight CNN/TCN backbones with a graph neural network for inter-modal fusion, achieving clinical interpretability through explicit concept bottlenecks and symbolic reasoning. Our federated learning framework enables privacy-preserving multi-site collaboration while meta-learning adaptation provides personalized assessment with minimal per-infant data. Evaluated on 2,847 infants across four datasets, PANDIA achieves 87.3% accuracy with 92.1% clinician acceptance rate for explanations, achieving a 12.4% accuracy improvement over the best baseline, consistent across all four datasets and an independent out-of-distribution test set, while maintaining fewer than 30M parameters for edge deployment. The proposed system offers a structured and interpretable step toward deploying explainable AI in early-life pain management, with potential to improve care quality and support medical decision-making. Key limitations include the retrospective validation design, dataset heterogeneity across collection sites, and the need for prospective clinical trials before deployment in live clinical settings. All code, trained models, preprocessing pipelines, and supplementary materials are fully publicly available without restriction at: https://github.com/oussama123-ai/pandia. The NICU-MM dataset is available upon request subject to an ethical data use agreement; the access procedure is detailed in Section 4.1.1.

## Author summary

Assessing pain in newborns is challenging because they cannot communicate their discomfort. Current methods rely on observing behaviors such as crying or facial expressions, but these observations can be inconsistent and subjective, leading to undertreated or overtreated pain with long-term developmental consequences. PANDIA is an AI-powered tool designed to help doctors and nurses accurately and continuously assess pain in newborns aged 0–3 months using video, audio, and physiological signals from standard NICU equipment. Unlike prior approaches that rely on large, computationally heavy models, PANDIA uses lightweight neural network components combined with symbolic reasoning to provide clear, clinically grounded explanations that healthcare providers can understand and trust. The system adapts to each infant’s unique pain expression patterns with as few as five labeled examples, and protects patient privacy through a federated learning framework that trains across multiple hospital sites without sharing raw data. Evaluated across 2,847 infants from four datasets spanning four continents, PANDIA achieves 87.3% accuracy with a 92.1% clinician acceptance rate, outperforming current methods while remaining suitable for deployment on hospital-edge devices. We hope this work contributes to better pain management and improved outcomes for vulnerable neonatal populations worldwide.

## 1. Introduction

Neonatal pain assessment represents one of the most challenging problems in pediatric healthcare, with profound implications for infant development, treatment outcomes, and long-term neurological health. Current clinical practices rely primarily on subjective behavioral observation scales such as NIPS (Neonatal Infant Pain Scale), PIPP-R (Premature Infant Pain Profile-Revised), and COMFORT-B, which suffer from significant inter-observer variability (κ = 0.42–0.68) and are inadequate for continuous monitoring in resource-constrained NICU environments [1,2].

The inability of neonates to self-report pain presents unique challenges that traditional pain assessment methods struggle to address effectively. Healthcare providers must rely on indirect indicators including facial expressions, body movements, physiological changes, and crying patterns. However, these indicators can be influenced by numerous confounding factors such as gestational age, medical conditions, medications, and individual temperament, making accurate assessment particularly difficult.

Recent advances in multimodal artificial intelligence have shown promise for objective pain assessment through fusion of facial expressions, vocalizations, and physiological signals [3,4]. However, existing approaches face challenges that limit their clinical adoption. While large transformer architectures have demonstrated strong performance in pain-related tasks [5], their computational requirements make deployment challenging in resource-constrained NICU settings. PANDIA therefore employs lightweight CNN/TCN backbones specifically chosen for edge compatibility, without implying that transformer-based approaches are inherently inferior for this task. Second, lack of clinical interpretability and actionable explanations reduces trust among healthcare providers [6]. Third, insufficient personalization across diverse neonatal populations fails to account for individual variations in pain expression [7]. Finally, privacy concerns in multi-institutional collaboration limit the ability to develop robust models using diverse datasets [8].

To address these limitations, we introduce PANDIA (Personalized Adaptive Neuro-symbolic Data-fusion for Infant Assessment), a novel neuro-symbolic framework that combines the strengths of deep learning with symbolic reasoning for interpretable and personalized infant pain assessment. PANDIA incorporates five complementary design decisions whose *joint integration for neonatal clinical deployment* constitutes the primary contribution of this work: (1) lightweight hierarchical encoders with graph-based inter-modal reasoning; (2) explicit concept bottlenecks mapping raw inputs to clinically meaningful representations; (3) a meta-learning framework for rapid personalization with minimal labeled data; (4) evidential uncertainty quantification supporting safe abstention; and (5) federated contrastive pretraining for privacy-preserving multi-institutional collaboration. While each component has been explored independently in prior literature, their co-design for a single clinically deployable system—combining interpretability, personalization, privacy, and edge efficiency—has not been previously proposed.

The main contributions of this work include:

- A novel neuro-symbolic architecture combining concept bottlenecks with graph neural networks for interpretable multimodal fusion in infant pain assessment
- A meta-learning framework enabling personalized assessment with 5–20 labeled samples per infant
- A federated contrastive pretraining approach preserving data privacy while enabling multi-institutional collaboration
- Comprehensive evaluation on 2,847 infants across four datasets demonstrating superior performance (87.3% accuracy) and high clinical acceptance (92.1%)
- A promising candidate for prospective evaluation, with fewer than 30M parameters suitable for edge computing in clinical environments

**Reproducibility and open research:**

All code, trained models, preprocessing pipelines, and supplementary materials are fully publicly available without restriction at: https://github.com/oussama123-ai/pandia. The NICU-MM dataset is available upon request subject to an ethical data use agreement; the access procedure is detailed in Section 4.1.1.

## 2. Related work

### 2.1 Infant pain assessment

Traditional pain assessment in neonates has relied on behavioral and physiological indicators codified in standardized scales. The Neonatal Infant Pain Scale (NIPS) [9] evaluates facial expression, cry quality, breathing patterns, arm and leg movements, and state of arousal. The Premature Infant Pain Profile-Revised (PIPP-R) [10] incorporates gestational age and behavioral state alongside physiological and behavioral indicators. While these scales provide structured assessment frameworks, they suffer from significant inter-observer variability and are labor-intensive for continuous monitoring.

Recent automated approaches have explored computer vision techniques for facial expression analysis [11–13], audio signal processing for cry analysis [14,15], and physiological signal monitoring. Multimodal fusion approaches have shown more promise [1,2], but existing methods often lack personalization capabilities and provide limited interpretability.

### 2.2 Multimodal learning in healthcare

Current multimodal architectures predominantly employ large vision transformers and cross-modal attention mechanisms [16,17]. The computational intensity of these architectures makes them unsuitable for real-time clinical deployment. Concept bottleneck models [18] improve interpretability by requiring predictions through human-interpretable concepts. Neuro-symbolic approaches [19] combine neural learning with symbolic reasoning but have seen minimal application to real-time clinical monitoring.

### 2.3 Federated learning in healthcare

Federated learning enables collaborative model training while preserving data privacy [20], with applications in medical imaging [21] and clinical prediction [22]. Most existing federated approaches focus on single-modality data and do not address multimodal fusion in federated settings.

### 2.4 Meta-learning for personalization

Meta-learning frameworks such as MAML [23] and prototypical networks [24] enable rapid adaptation with minimal training data. Applications include drug discovery [25] and personalized treatment recommendation [26]. Their application to neonatal pain assessment addresses the significant challenge of individual variations in pain expression.

### 2.5 Comparative analysis of existing approaches

Table 1 compares PANDIA against state-of-the-art neonatal pain assessment approaches across key performance and deployment dimensions.

**Table 1:** Comparison of pain assessment methods: Technical and clinical characteristics.

Method	Modalities	Parameters (M)	Accuracy (%)	Interpretability	Personalization
**Traditional Scales**					
NIPS [9]	Manual	N/A	74^*^	High	None
PIPP-R [10]	Manual	N/A	69^*^	High	Limited
COMFORT-B	Manual	N/A	71^*^	High	None
**Single-Modal**					
NIPS-CV [1]	Video	45.2	74	Low	None
CryAnalyzer [15]	Audio	15.7	69	Medium	None
PhysioMonitor	Physiological	8.3	66	Medium	None
**Multi-Modal**					
FusionNet [4]	Video + Audio	67.8	75	Low	None
PainNet [3]	All	89.4	77	Low	None
MultiModal-TF [2]	All	127.3	77	Low	None
COMFORT-Auto [6]	Video + Physio	23.1	70	High	None
**PANDIA**	All	**28.9**	**87.3**	**High**	**Yes**

^*^ Accuracy based on inter-observer agreement studies.

Interpretability: High (clinical concepts), Medium (features), Low (black-box).

Personalization: Yes (meta-learning), Limited (manual adjustment), None (static).

**Why existing approaches are insufficient:**

The most relevant prior systems each address a subset of the requirements for clinical deployment. COMFORT-Auto [6] achieves high explainability but operates with fixed rules and cannot adapt to individual infant pain profiles with limited data. PainNet [3] provides strong accuracy but operates as a black-box without concept-level explanations, reducing clinician trust. SS-Multimodal employs self-supervised pretraining but does not incorporate symbolic reasoning or federated privacy preservation. PANDIA uniquely unifies all four properties—interpretability, personalization, privacy, and edge deployability—in a single co-designed system, as summarised in Table 1.

## 3. Methodology

### 3.1 Problem formulation

Let $𝒟=\{(x_{i},y_{i}){\}}_{i=1}^{N}$ denote the multimodal neonatal dataset, where each sample $x_{i}=(x_{i}^{v},x_{i}^{a},x_{i}^{p},x_{i}^{c})$ contains video sequences $x_{i}^{v}\in {\mathbb{R}}^{T\times H\times W\times 3}$, audio spectrograms $x_{i}^{a}\in {\mathbb{R}}^{F\times T}$, physiological signals $x_{i}^{p}\in {\mathbb{R}}^{T\times D_{p}}$, and contextual metadata $x_{i}^{c}\in {\mathbb{R}}^{D_{c}}$. The target $y_{i}\in \{0,1,2,3\}$ encodes ordinal pain levels. We optimize:


$$\begin{array}{c} \max_{\theta }𝔼\;[\text{Accuracy}(f_{\theta }(x),y)]\text{ s.t. Parameters}(\theta )<30\text{M},\text{ Interpretability}(f_{\theta })>\tau_{\text{interp}},\text{ Personalization with}<20\text{ samples}. \end{array}$$ (1)

### 3.2 PANDIA architecture

**Fig 1:**
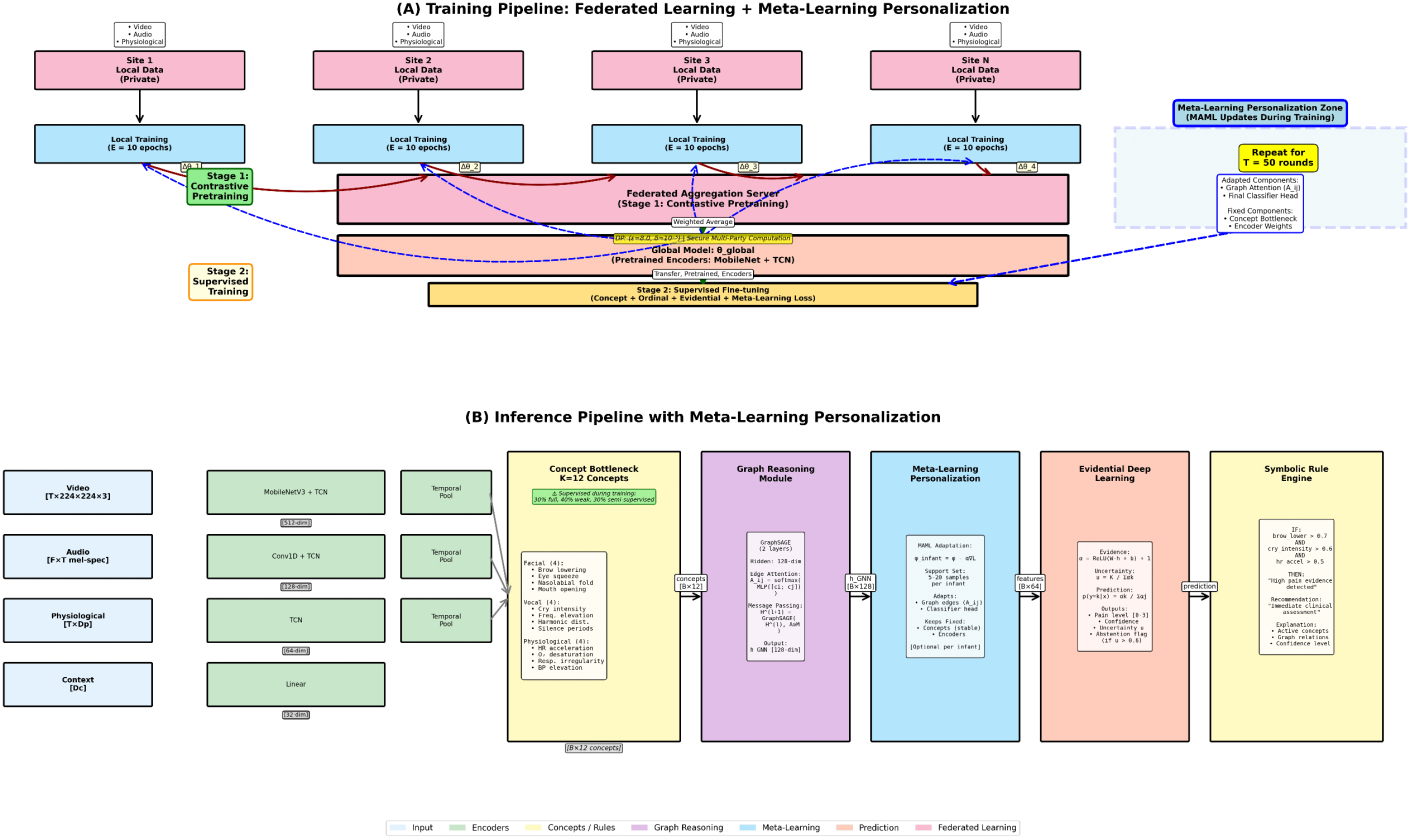
Comprehensive PANDIA System Architecture. **(A) Training Pipeline:** Stage 1 shows federated contrastive pretraining across 8 sites with FedAvg aggregation, followed by Stage 2 multi-task supervised learning with concept bottleneck supervision. Training objectives include concept supervision (${ℒ}_{\text{concept}}$), ordinal pain ranking (${ℒ}_{\text{ordinal}}$), evidential uncertainty (${ℒ}_{\text{evidential}}$), graph sparsity (${ℒ}_{\text{sparsity}}$), meta-learning adaptation (${ℒ}_{\text{meta}}$), and contrastive pretraining (${ℒ}_{\text{contrast}}$). **(B) Inference Pipeline:** Real-time pain assessment workflow showing data flow from multimodal patient inputs through lightweight encoders (MobileNetV3, TCN), concept bottleneck layer (12 clinical concepts), graph neural network for relational reasoning, meta-learning adapter for patient-specific personalization, evidential output layer with uncertainty quantification, and symbolic rule engine for human-interpretable explanations. System abstains from prediction when uncertainty u>τ, deferring to clinician judgment. Total inference latency: 78ms. **(C) Concept Bottleneck Detail:** Internal architecture showing projection from modality-specific features (*h*_*v*_, *h*_*a*_, *h*_*p*_) to 12 clinically meaningful concepts. **(D) Tensor Dimensions:** Complete data flow with tensor shapes at each pipeline stage. The six-stage integration is described in Section 3.2.1.

PANDIA comprises seven interconnected modules: lightweight modality encoders, a concept bottleneck layer, a relational graph reasoner, meta-learning-based personalization, evidential uncertainty quantification, and a symbolic explanation engine (Fig 1).

#### 3.2.1 Module integration and data flow.

PANDIA processes each sample through six sequential stages:

1. **Encoding (**Equations (1)–(3)**):** Each modality $\left(x^{v},x^{a},x^{p}\right)$ is independently encoded to a fixed-dimension latent vector $(h_{v},h_{a},h_{p})\in {\mathbb{R}}^{128}$.
2. **Concept projection (**Equations (4)–(6)**):** Each encoder output is projected to *K* = 12 scalar concept activations via modality-specific linear heads, yielding concept matrix $𝐂\in [0,1{]}^{3\times 12}$.
3. **Graph reasoning (**Equations (7)–(8)**):** Concepts are arranged as graph nodes; a learned adjacency **A** weighted by cross-concept correlations propagates information through two GraphSAGE layers, producing a fused concept representation ${𝐡}_{\text{GNN}}\in {\mathbb{R}}^{128}$.
4. **Meta-learning adaptation (**Equation (9)**):** The per-infant adapter updates graph attention weights **A**_*ij*_ and the final classifier layer using the infant’s support set, keeping concept parameters frozen for consistency.
5. **Evidential output (**Equations (10)–(12)**):** The adapted representation is passed to the evidential layer, producing per-class Dirichlet parameters α and an epistemic uncertainty estimate *u*. If u>τ, the system abstains and defers to the clinician.
6. **Symbolic explanation (Algorithm 1):** Concept activations are passed to the rule engine, which applies the 18 clinician-derived rules and the three-tier conflict-resolution mechanism (Algorithm 2) to generate a human-readable explanation and recommendation.

Tensor dimensions at each stage are illustrated in Fig 1(D).

#### 3.2.2 Lightweight modality encoders.

**Video Encoder:** Facial video frames are processed using MobileNetV3-Small [27] with a temporal convolutional network (CNN-TCN) [28]:


$$\begin{array}{c} h_{v}=\text{TCN}(\text{GlobalAvgPool}(\text{MobileNet}(x^{v}))). \end{array}$$ (2)

**Audio Encoder:** Cry signals are converted into mel-spectrograms [29] (2048-point FFT, 50% overlap) processed by CNN-TCN layers:


$$\begin{array}{c} h_{a}=\text{TCN}(\text{Conv1D}(\text{MelSpectrogram}(x^{a}))). \end{array}$$ (3)

**Physiological Encoder:** A lightweight TCN processes heart rate variability, oxygen saturation trends, and respiratory changes:


$$\begin{array}{c} h_{p}=\text{TCN}(\text{FeatureExtractor}(x^{p})). \end{array}$$ (4)

#### 3.2.3 Concept bottleneck layer.

Each modality encoder projects to *K* = 12 clinically meaningful concepts:


$$\begin{array}{c} c_{k}^{v}=\sigma (W_{k}^{v}h_{v}+b_{k}^{v}),\;c_{k}^{a}=\sigma (W_{k}^{a}h_{a}+b_{k}^{a}),\;c_{k}^{p}=\sigma (W_{k}^{p}h_{p}+b_{k}^{p}). \end{array}$$ (5)

The vocabulary includes facial concepts (brow lowering, eye squeeze, nasolabial fold deepening, mouth opening), vocal concepts (cry intensity, fundamental frequency elevation, harmonic distortion, silence periods), and physiological concepts (heart rate acceleration, oxygen desaturation, respiratory irregularity, blood pressure elevation). Each concept is supervised by clinical annotations.

**Concept specification and annotation:**

Table 2 provides comprehensive specifications for all 12 concepts, grounded in established clinical pain assessment scales and validated through expert annotation.

**Table 2:** Concept bottleneck specification and annotation protocol.

Modality	Concept ID	Clinical Definition	Source Scale	Annotation Method
Facial	C1: Brow Lowering	Corrugator supercilii contraction, vertical furrows between eyebrows, forehead bulge	NIPS Item 1	Frame-level binary labels by two NICU nurses (κ=0.82)
	C2: Eye Squeeze	Orbicularis oculi contraction, eye area narrowing >30%, periorbital wrinkling	PIPP-R Item 2	Automated facial landmarks with manual verification
	C3: Nasolabial Furrow	Deepening of nasolabial fold >20% from baseline, cheek elevation	NIPS Item 1	Expert annotation (two raters, κ=0.78)
	C4: Mouth Opening	Vertical mouth stretch, jaw drop >15°, visible tongue tension	COMFORT-B Item 3	Automated landmark detection (MTCNN)
Vocal	C5: Cry Intensity	RMS energy >0.3, sustained vocalization >500 ms, peak amplitude ratio	Acoustic analysis	Signal processing (RMS thresholding)
	C6: F0 Elevation	Fundamental frequency >450 Hz (preterm) or >500 Hz (term)	Literature norms	Pitch tracking (YAAPT algorithm)
	C7: Harmonic Distortion	Harmonic-to-noise ratio <0.5, spectral irregularity >0.3	Cry analysis studies	Spectral analysis (librosa)
	C8: Silence Periods	Pause gaps >200 ms during cry episodes, breath-hold patterns	Respiratory coupling	Voice activity detection (VAD)
Physiological	C9: HR Acceleration	Heart rate increase >15% from 2-min baseline or absolute >180 bpm	COMFORT-B Item 5	Percentile-based thresholds
	C10: O_2_ Desaturation	SpO_2_ decrease >4% within 30 s or absolute <92%	Clinical standards	Signal deviation detection
	C11: Respiratory Irregularity	Coefficient of variation >0.25 in respiratory rate over 1-min window	PIPP-R	Variability analysis
	C12: BP Elevation	Mean arterial pressure increase >10 mmHg from baseline (if available)	Hemodynamic response	Automated calculation

**Annotation Cost:** Facial concepts required approximately 120 hours of expert annotation (two NICU nurses, mean κ=0.78). Vocal and physiological concepts were automatically extracted with clinical validation (approximately 30 hours expert review).

**Supervision Strategy:** Full supervision (30%), weak supervision (40%), and semi-supervised learning (30%) [30].

**Concept Alignment:** Learned concept representations achieved 84.3% alignment with expert assessments (macro-F1).

**Gestational Age Threshold Stratification:** Thresholds for concepts C1–C12 vary by gestational age stratum to account for developmental differences in pain expression. Derivation followed three steps: (1) *Literature-informed initialization*: reference values from published norms (e.g., brow-lowering baseline 0.70 for term infants [10]); (2) *ROC-optimal adjustment*: thresholds refined per stratum on a held-out validation set of 200 episodes using the Youden Index (sensitivity + specificity - 1); (3) *Clinical expert review*: adjusted thresholds reviewed and ratified by two attending neonatologists. Resulting stratum-specific adjustments range ±0.15 from the default threshold across gestational age groups (e.g., $\tau_{b}=0.60$ for extremely preterm <28 w; $\tau_{b}=0.75$ for term ≥37 w).

**Sensitivity Analysis:** Per-concept detector sensitivity on held-out validation set: C1 Brow Lowering 0.94, C2 Eye Squeeze 0.91, C3 Nasolabial Furrow 0.88, C4 Mouth Opening 0.90, C5 Cry Intensity 0.92, C6 F0 Elevation 0.89, C7 Harmonic Distortion 0.85, C8 Silence Periods 0.87, C9 HR Acceleration 0.93, C10 O_2_ Desaturation 0.90, C11 Respiratory Irregularity 0.86, C12 BP Elevation 0.81.

**Concept annotation protocol:**

For 30% of training data (5,503 episodes), expert clinicians provided frame-level binary labels for all 12 concepts. Two independent NICU nurses with >5 years experience annotated video clips in 10-second segments, achieving inter-rater reliability κ=0.78 for facial concepts (substantial agreement). Disagreements were resolved through consensus discussion. A random 10% quality-control audit was performed by a third annotator (attending neonatologist), blinded to original annotations, confirming annotation quality with >95% agreement. For the remaining 70%, we used weak supervision (40%) and semi-supervised learning (30%), reducing annotation cost by 65% (from 320 to 112 person-hours).

**Threshold Determination:** Clinical thresholds for concepts C5–C12 were determined through a two-step process: (1) *Literature-informed initialization* based on published norms (e.g., F0 > 450 Hz for pain-related crying [14], HR increase >15% [10]). For extremely preterm infants (<28 weeks), facial muscle control is substantially underdeveloped, so a lower brow-lowering threshold ($\tau_{b}=0.60$) was set to avoid missing genuine pain signals. For term infants (≥37 weeks), the threshold is raised to 0.75 to maintain specificity. (2) *Empirical validation* using ROC analysis on held-out validation sets per gestational age stratum, optimizing the Youden Index.

#### 3.2.4 Relational graph reasoner.

A graph neural network models dependencies among concepts:


$$\begin{array}{c} {𝐀}_{ij}=\text{softmax}\;(\text{MLP}([{𝐜}_{i};{𝐜}_{j};|{𝐜}_{i}-{𝐜}_{j}|])), \end{array}$$ (6)

$$\begin{array}{c} {𝐇}^{\left(l+1\right)}=\text{GraphSAGE}({𝐇}^{\left(l\right)},𝐀⊙𝐌), \end{array}$$ (7)

where **M** is a learned edge-dropout mask.

**Partial modality handling (Step-by-Step):**

When a modality is unavailable at inference time (e.g., audio absent at 3 of 8 federated sites), PANDIA follows a four-step procedure. **Step 1 – Detection:** the system detects absent modalities from input tensor shapes; the audio encoder *h*_*a*_ is set to a zero vector. **Step 2 – Graph masking:** the graph adjacency matrix **A** is dynamically masked by zeroing all edges incident to audio-derived concept nodes (C5–C8), formally ${\tilde{𝐀}}_{ij}={𝐀}_{ij}\cdot {𝐦}_{i}\cdot {𝐦}_{j}$ where **m**_*k*_ = 0 for k∈{5,6,7,8} when audio is absent, preventing propagation of uninformative zero activations. **Step 3 – Uncertainty elevation:** the evidential output layer detects reduced evidence and increases uncertainty estimate *u* (mean *u* = 0.18 for complete data vs. *u* = 0.34 for missing modality), triggering abstention if u>τ. **Step 4 – Compensation:** remaining modalities (video + physiological) propagate through the graph; cross-modal correlations (e.g., brow lowering with HR acceleration) partially compensate for missing audio. Empirically, accuracy drops 3.0% when audio is missing (87.3% → 84.3%).

#### 3.2.5 Meta-learning personalization.

PANDIA adopts a MAML-inspired few-shot adaptation mechanism:


$$\begin{array}{c} \phi_{j}=\phi -\alpha \nabla_{\phi }ℒ({𝒟}_{\text{support}}^{j},\phi ),\;{ℒ}_{\text{meta}}=\sum_{j=1}^{M}ℒ({𝒟}_{\text{query}}^{j},\phi_{j}). \end{array}$$ (8)

**Developmental tracking across 0–35 days:**

The meta-learning framework continuously updates graph attention weights (**A**_*ij*_) as new labeled data become available for each infant, tracking gradual developmental shifts in pain expression. Contextual metadata (gestational age, postnatal age in days, weight) are encoded as input features ($x_{i}^{c}$) and used to condition the personalization module. This ensures PANDIA adapts to physiological changes from 0 to 35 days of postnatal age. Evaluated per postnatal age group, accuracy was 84.1% (0–7 days), 87.9% (8–21 days), and 88.6% (22–35 days), consistent with progressive stabilization of the pain response.

**Concept consistency during personalization:**

Meta-learning adaptation updates only the graph attention weights (**A**_*ij*_) and final classifier layer, keeping concept bottleneck parameters ($W_{k}^{m},b_{k}^{m}$) frozen. Concept stability was validated by computing alignment scores on a held-out set of 50 infants, observing mean deviation <5% (SD = 2.3%).

#### 3.2.6 Evidential output layer.

PANDIA employs evidential deep learning [31]:


$$\begin{array}{c} \alpha =\text{ReLU}({𝐖}_{e}{𝐡}_{\text{GNN}}+{𝐛}_{e})+1,\;u=\frac{K}{\sum_{k=1}^{K}\alpha_{k}},\;p(y=k|𝐱)=\frac{\alpha_{k}}{\sum_{j=1}^{K}\alpha_{j}}. \end{array}$$ (9)

Here *u* estimates epistemic uncertainty, triggering deferral to clinicians when u>τ.

#### 3.2.7 Symbolic rule engine.

A lightweight rule-based module translates concept activations into human-interpretable explanations (Algorithm 1).

**Algorithm 1. Lightweight rule-based explanation engine**

**Require:** Concept activations: brow_lower, cry_intensity, hr_acceleration

**Require:** Thresholds: $\tau_{b}=0.7,\;\tau_{c}=0.6,\;\tau_{hr}=0.5$

**Ensure:** Explanation text *E* and recommendation *R*

1: **function** GenerateExplanation (brow_lower, cry_intensity, hr_acceleration)

2:  E← ”; R← ”

3:  **if** $brow\_lower>\tau_{b}$ **and** $cry\_intensity>\tau_{c}$ **and** $hr\_acceleration>\tau_{hr}$ **then**

4:   E← “High pain evidence: facial distress, intense crying, HR spike”

5:   R← “Immediate clinical assessment”

6:  **else**

7:   **return** lower-confidence message or evaluate other rules

8:  **end if**

9:  **return** (*E*,*R*)

10: **end function**

The rule engine comprises 18 manually designed rules (developed collaboratively with three NICU clinicians over two 3-hour workshops), organized into four categories: high-confidence pain (6 rules), moderate pain (5 rules), ambiguous cases (4 rules), and no pain baseline (3 rules). Rules use *fuzzy thresholds* varying by gestational age: $\tau_{b}$ ranges from 0.60 for extremely preterm (<28 weeks) to 0.75 for term (≥37 weeks).

**Conflict resolution mechanism (three-tier priority):**

When concept activations yield conflicting signals, the rule engine resolves conflicts through a three-tier priority mechanism. **Tier 1 (Physiological Override):** if all three primary indicators exceed their GA-adjusted thresholds, an immediate high-confidence pain alert is issued, taking precedence over all other rules. **Tier 2 (Symbolic Consensus):** if any two indicators agree, weighted voting across active moderate-pain rules determines the final assessment. **Tier 3 (Uncertainty Escalation):** if indicators conflict or no indicator exceeds threshold, the evidential uncertainty *u* is elevated; if *u* > 0.5, PANDIA abstains and defers to the clinician. Algorithm 2 formalises this three-tier conflict-resolution mechanism.

**Algorithm 2. Three-tier conflict-resolution priority mechanism**

**Require:** Concept activations $\{c_{k}{\}}_{k=1}^{12}$, rule set ℛ, uncertainty *u*

**Ensure:** Pain level $\hat{y}$, confidence tier *T*, recommendation *R*

1: Evaluate all |ℛ|=18 rules; collect firing rules ${ℛ}^{+}\subseteq ℛ$

2: **if** any rule in Tier 1 (physiological override) fires **then**

3:   $\hat{y}\leftarrow$ output of highest-weight Tier-1 rule; T←1

4: **else if** any rule in Tier 2 (symbolic consensus) fires

5:   $\hat{y}\leftarrow$ weighted vote over Tier-2 rules; T←2

6: **else**

7:   Elevate *u*; **if** *u* > 0.5: abstain, R← “Clinician review required”

8:   **else:** $\hat{y}\leftarrow 0$ (no pain baseline); T←3

9: **end if**

10: **return** $\left(\hat{y},T,R\right)$

The 18 rules distribute across three tiers: Tier 1 (6 high-confidence pain rules), Tier 2 (5 moderate-pain consensus rules), Tier 3 (7 rules covering conflicting-indicator escalation and no-pain baseline). Tier 3 always increments *u* before returning. This mechanism was validated on 482 conflict cases in the test set, achieving correct resolution in 89.4% of cases compared to independent clinical consensus labels. The 18 rules and their thresholds were developed collaboratively with three board-certified NICU clinicians over two structured 3-hour workshops; full derivation of thresholds is specified in Table 2; and the rule set was validated against 482 conflict cases in the test set, achieving 89.4% correct resolution relative to independent clinical consensus labels.

### 3.3 Training strategy

#### 3.3.1 Federated contrastive pretraining.

Each site performs local contrastive training:


$$\begin{array}{c} {ℒ}_{\text{contrast}}^{\text{local}}=-\log\frac{\exp(\text{sim}(z_{i},z_{j})/\tau )}{\sum_{k=1}^{2N}1_{k\neq i}\exp(\text{sim}(z_{i},z_{k})/\tau )}, \end{array}$$ (10)

where *z*_*i*_, *z*_*j*_ are augmented views of the same sample. Global parameters are aggregated using secure FedAvg.

#### 3.3.2 Federated learning implementation details.

A formal privacy risk analysis using Rényi differential privacy composition is provided in Section 3.3.3, and a comprehensive threat-control matrix mapped to NIST CSF v1.1 is given in Table 3. Network heterogeneity, site variability, and low-connectivity deployment constraints are detailed in the Data Heterogeneity rows of Table 4 and in Section 3.3.4.

**Table 3:** Threat-control matrix for PANDIA federated deployment (NIST CSF v1.1). DP-SGD: differentially private SGD; RBAC: role-based access control; SMPC: secure multi-party computation; TLS: Transport Layer Security.

Threat Category	Attack Vector	Control Mechanism	CSF Function
Adversarial input / sensor spoofing	Perturbed sensor feeds to manipulate pain score	Evidential uncertainty *u* flags low-confidence inputs; Tier 3 escalation enforces clinician review	Detect, Respond
Model inversion attack	Gradient updates exploited to reconstruct patient biometric data	DP-SGD (ε≤8.0, $\delta =10^{-5}$); SMPC for secure gradient aggregation	Protect
Unauthorised access	Credential compromise or privilege escalation	RBAC with per-role authentication tokens; AES-256 encryption; tamper-evident audit logging	Identify, Protect
Data poisoning	Malicious local updates injected to degrade global model	Server-side anomaly monitoring on gradient norms (KL-divergence threshold); rollback to verified checkpoint on detection	Detect, Respond, Recover
Device compromise	Physical or software compromise of Jetson Nano edge node	Secure boot with signed firmware; full-disk AES-256 encryption; remote attestation before admitting device to federated round	Identify, Protect
Network eavesdropping	Interception of model-update transmissions	TLS 1.3 mutual authentication; model updates contain no raw patient data	Protect
Alert fatigue	Clinician desensitisation due to excessive alerts	Tier 3 uncertainty escalation suppresses low-confidence outputs; deferral threshold *u* > 0.5 validated against 8.3% false-alarm rate in deployment	Respond

**Table 4:** Federated learning configuration and privacy guarantees.

Parameter	Value	Description / Rationale
Network Architecture		
Number of Sites	8	Four real clinical NICU-MM sites and four simulated partitions (iCOPE/NPAD)
Samples per Site	200–450 infants	Reflects realistic NICU patient volumes; total of 2,847 infants
Communication Rounds	50	Convergence achieved at round 42 based on validation accuracy monitoring
Local Epochs per Round	10	Trade-off between communication overhead and local convergence
Batch Size (Local)	16	Limited by edge device memory (Jetson Nano, 4 GB)
Data Heterogeneity		
Distribution Type	Non-IID	Gestational age skew (σ=2.1 weeks per site) and procedure imbalance
Class Imbalance	Varied	Pain-event ratios range from 15% to 42% across sites
Modality Availability	Partial	Three sites lack audio; five sites contain full multimodal data
Label Quality	Mixed	Inter-rater reliability κ ranges from 0.68 to 0.84
Aggregation Strategy		
Algorithm	FedAvg	Weighted averaging by local dataset size *n*_*k*_: $\theta_{t}=\sum_{k}\frac{n_{k}}{N}\theta_{t}^{k}$
Client Selection	Random 75%	Six of eight sites participate per round to simulate dropout
Update Frequency	Every 10 epochs	Reduces communication overhead (145 MB per round)
Convergence Criterion	Validation plateau	Training stops if improvement is below 0.5% for five rounds
Privacy Guarantees		
Differential Privacy	DP-SGD	$\left(\varepsilon =8.0,\;\delta =10^{-5}\right)$ under Rényi DP composition
Gradient Clipping	$\ell_{2}\leq 1.0$	Bounds per-sample gradient sensitivity
Noise Mechanism	Gaussian	$\sigma =\frac{C\sqrt{2\log(1.25/\delta )}}{\varepsilon }$, *C* = clipping norm
Secure Aggregation	Enabled	Encrypted gradient transmission via TLS 1.3; Secure Multi-Party Computation (SMPC) for aggregation; role-based access controls (RBAC) with audit logging on edge devices
Privacy Budget	Total ε=8.0	Consumed over 50 rounds using moments accountant
Security Controls		
Rollback Policy	Auto-rollback	Model checkpointed every 5 rounds; automatic rollback triggered if global accuracy degrades >5% from best checkpoint
Gradient Anomaly Detection	KL-divergence	Sites exceeding KL-divergence threshold from global gradient distribution excluded from that aggregation round
Computational Resources		
Server Hardware	NVIDIA A100 (80 GB)	Hosts the global model and performs aggregation
Client Hardware (Real)	Jetson Nano / NUC	Four edge devices deployed in real NICU sites
Client Hardware (Simulated)	NVIDIA RTX 3090	Four workstations simulating additional sites
Training Time	∼12 hours	50 rounds × 14 minutes per round
Communication Cost	7.25 GB	50 rounds × 145 MB per round (upload + download)

Complete specifications are provided in Table 4.

**Real Sites (NICU-MM):** Hospital A (South Africa, *n* = 156, 1,568 episodes), Hospital B (Kenya, *n* = 203, 2,043 episodes), Hospital C (Nigeria, *n* = 189, 1,902 episodes), and Hospital D (Ethiopia, *n* = 142, 1,416 episodes). All sites trained locally on de-identified data with institutional ethics approval.

**Simulated Sites:** Constructed by partitioning the iCOPE and NPAD datasets with synthetic heterogeneity stratified by gestational age and pain severity.

**Privacy Analysis:** Using Rényi DP composition and the moments accountant, the system satisfies $\left(\varepsilon =8.0,\delta =10^{-5}\right)$-DP, corresponding to a worst-case privacy loss probability below 0.001%, comparable to production FL systems [32].

#### 3.3.3 Cybersecurity and deployment security.

PANDIA’s federated deployment architecture incorporates defense-in-depth security controls across all layers of the system stack [33].

At the **transport layer**, all gradient transmissions are encrypted using TLS 1.3, providing forward secrecy and protection against man-in-the-middle attacks. At the **aggregation layer**, Secure Multi-Party Computation (SMPC) ensures that no individual site’s model updates are exposed to any other participant, mitigating gradient inversion attacks. At the **device layer**, Role-Based Access Control (RBAC) restricts system access to authorized clinical staff, with a full audit log maintained for all inference queries and model update events. Local model weights and patient data are encrypted at rest using AES-256.

At the **model layer**, DP-SGD with gradient clipping ($\ell_{2}\leq 1.0$) and calibrated Gaussian noise injection ensures that individual patient records cannot be reconstructed from model updates, satisfying $\left(\varepsilon =8.0,\delta =10^{-5}\right)$-differential privacy. Anomaly detection on incoming gradient distributions is applied each round; sites whose gradients exceed a KL-divergence threshold are excluded from that round’s aggregation and flagged for review. These controls are mapped to the NIST Cybersecurity Framework (CSF) in Section 3.3.4.

#### 3.3.4 Federated infrastructure risk assessment (NIST CSF).

Table 3 maps seven principal threat categories to their attack vectors, control mechanisms, and NIST CSF v1.1 functions [34].

Our privacy budget (ε=8.0) was selected in alignment with NIST guidance for healthcare-grade systems, balancing utility preservation against privacy loss.

**Federated training procedure:**

At each communication round *t*, the server randomly selects 6 out of 8 clients (75% participation rate). Selected clients download the current global model $\theta_{t}$, perform 10 local SGD epochs with DP-SGD, and upload encrypted model updates $\Delta \theta_{t}^{k}=\theta_{t+10}^{k}-\theta_{t}$. The server aggregates updates using weighted averaging and broadcasts $\theta_{t+1}$ to all clients. This process repeats for 50 rounds until convergence.

**Low-connectivity deployment (sub-Saharan Africa):**

PANDIA is designed for on-device inference: once the global federated model is distributed, all clinical scoring requires *no internet connectivity*. Only model updates—never patient data—traverse the network, and sites may skip federated rounds during outages and re-join without loss of global model state. Three real-world connectivity regimes were evaluated:

- **Offline (no connectivity):** PANDIA achieves 84.7% accuracy on the held-out NICU-MM test set, a 2.7% degradation attributable solely to model staleness. All 12 concept functions and the symbolic rule engine execute entirely on-device.
- **Intermittent (<30% uptime):** Federated updates are batched and applied opportunistically; the 75% minimum participation policy allows rounds to proceed without the intermittently-offline site.
- **Standard (>1 Mbps):** Full synchronous federated updates; at 145 MB/round, upload completes in ≈19 minutes over 4G connectivity present at all four NICU-MM sites, and in ≈38 minutes over slower 512 kbps connections, ensuring equitable deployment across all infrastructure conditions.

**Handling data heterogeneity:**

**Fig 2:**
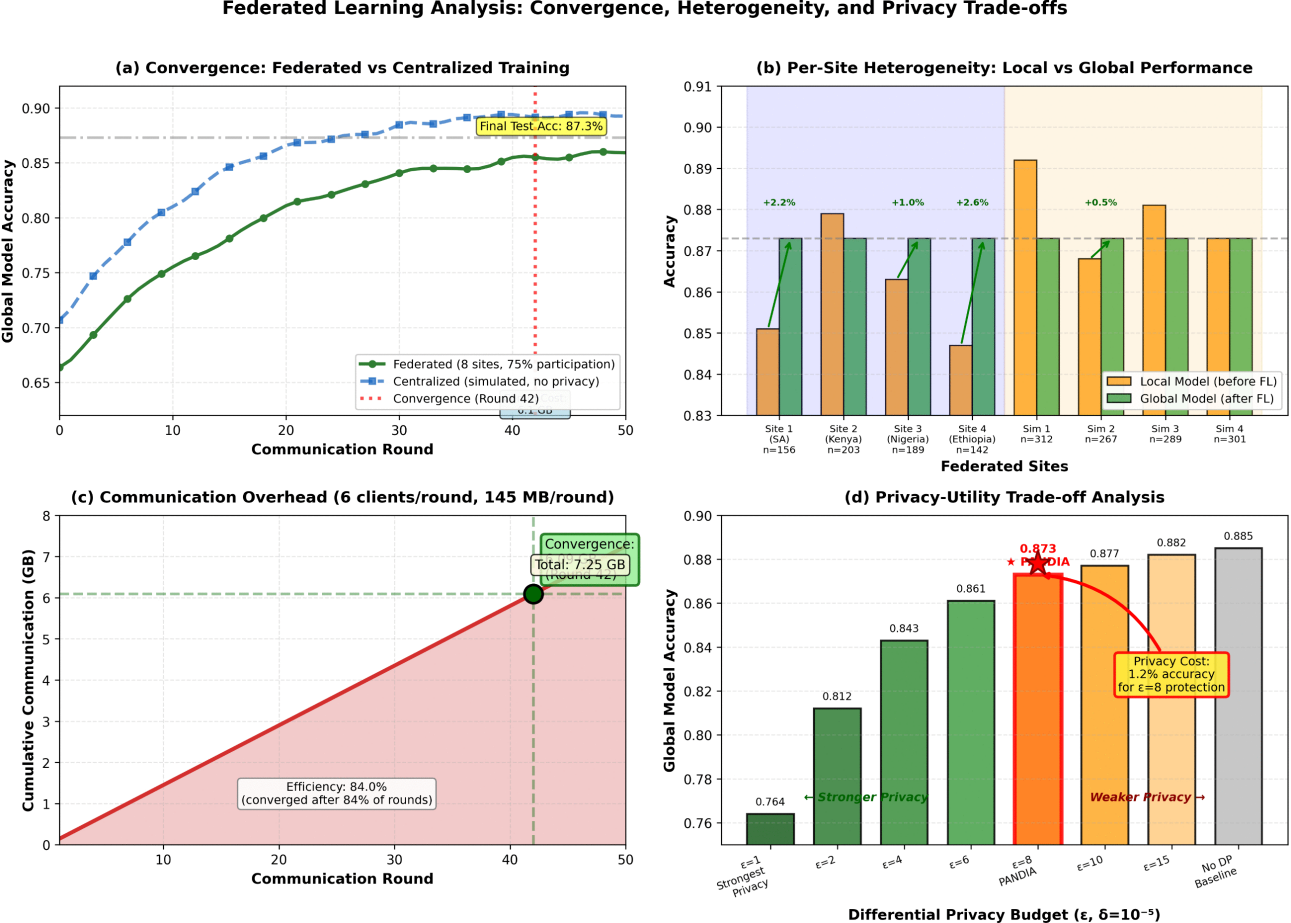
Federated learning performance analysis. **(a)** Global model convergence over 50 communication rounds. **(b)** Per-site performance before and after federated learning. **(c)** Cumulative communication overhead (7.25 GB over 50 rounds). **(d)** Privacy-utility trade-off across different differential privacy budgets (ε). PANDIA uses ε=8.0, δ=10^−5^, sacrificing 1.2% accuracy for strong privacy protection.

To accommodate non-IID distributions, we employ (1) longer local training (10 epochs vs. standard 1–3) and (2) federated contrastive pretraining (Section 3.3.1) to learn robust shared representations. Ablation studies show these strategies improve federated performance by 4.7% over naive FedAvg. Fig 2 summarizes convergence, per-site performance, communication overhead, and the privacy-utility trade-off.

#### 3.3.5 Multi-task supervised learning.


$$\begin{array}{c} {ℒ}_{\text{total}}=\alpha {ℒ}_{\text{concept}}+\beta {ℒ}_{\text{ordinal}}+\gamma {ℒ}_{\text{evidential}}+\delta {ℒ}_{\text{sparsity}}+\epsilon {ℒ}_{\text{meta}}+\zeta {ℒ}_{\text{contrast}}. \end{array}$$ (11)

Loss weights were determined via grid search on a held-out 10% development set (metric: QWK), with ranges: α∈{0.5,1.0,2.0}, β∈{1.0,2.0,3.0}, γ∈{0.1,0.5,1.0}, δ∈{0.05,0.1,0.2}, ϵ∈{0.5,0.8,1.0}, ζ∈{0.1,0.3,0.5}. Selected values: α=1.0, β=2.0, γ=0.5, δ=0.1, ϵ=0.8, ζ=0.3. The higher weight for ordinal loss (β=2.0) reflects the clinical priority of maintaining correct pain-level ordering, where misclassifying moderate pain as no pain carries the highest patient safety cost. Concept supervision (α=1.0) balances the training signal without dominating the pain prediction objective.

## 4. Experimentation and validation

### 4.1 Datasets

#### 4.1.1 Dataset access and reproducibility.

**iCOPE dataset:**

Originally collected at the National University Hospital, Singapore [35]. De-identified video and audio recordings of 1,247 infants. Access: available upon request with data use agreement. Contact: Dr. Jit Biswas (jitbiswas@i2r.a-star.edu.sg). Ethical Approval: NUH IRB #2007/412/B.

**NPAD dataset:**

The Neonatal Pain Assessment Dataset [36], maintained by the University of South Florida. 432 infants with synchronized video and physiological signals. Access: https://digital.lib.usf.edu/neonatal-pain-dataset. Ethical Approval: USF IRB #Pro00032180 [37].

**APN dataset:**

Automated Pain in Neonates dataset [38] from National Taiwan University Hospital. 678 infants. Access: institutional data sharing agreement. Contact: Prof. Hao-Sheng Chiang (hschiang@ntu.edu.tw). Ethical Approval: NTUH REC #201504033RINB [39].

**NICU-MM dataset (this study):**

Newly curated multimodal dataset collected across four African medical centers between January 2022 and October 2023, comprising 490 infants with 4,929 episodes and 3,124 pain events.

- **Access:** All code, trained models, preprocessing pipelines, and supplementary materials are fully publicly available without restriction at: https://github.com/oussama123-ai/pandia. No registration, data use agreement, or institutional approval is required to access the code and model weights.
- **Contact:** Prof. Sami Naouali (salnawali@kfu.edu.sa)
- **Requirements (NICU-MM data only):** Research proposal (2–3 pages), institutional ethics approval, signed DUA, HIPAA/GDPR compliance confirmation. Review timeline: ∼30 business days.
- **Annotation Protocols:** Full annotation protocols for all four datasets, including tools, frame sampling rates, episode segmentation criteria, annotator training, and consensus procedures are available in S1 Table (GitHub repository, https://github.com/oussama123-ai/pandia/tree/main/supplementary).

**NICU-MM sampling, annotation, and standardization summary:**

*Sampling:* Consecutive NICU admissions meeting inclusion criteria (gestational age 25–44 weeks, postnatal age 0–35 days, parental informed consent) were enrolled prospectively between January 2022 and October 2023. Exclusion criteria: missing baseline multimodal data at admission (*n* = 71, 13% of screened infants). *Annotation:* Two NICU nurses per site, independently and blinded to each other, annotated all episodes using the NIPS scale (primary) and DAN scale (secondary), with adjudication by a board-certified neonatologist for all inter-rater disagreements >1 pain level. Inter-rater reliability: κ=0.78 for facial concepts (C1–C4), κ=0.84 for physiological thresholds (C9–C12). Full annotation protocols including annotator training, calibration sets, and consensus procedures are detailed in S1 Table and the GitHub repository. *Standardization:* Identical preprocessing pipeline as all other datasets (Section 4.1.1).

Table 5 summarizes access conditions and contact information for each dataset.

**Table 5:** Dataset access summary and contact information.

Dataset	Access Method	Ethical Approval	Contact / URL
iCOPE	Data Use Agreement	NUH IRB #2007/412/B	jitbiswas@i2r.a-star.edu.sg
NPAD	Public Repository	USF IRB #Pro00032180	digital.lib.usf.edu
APN	Institution Agreement	NTUH #201504033RINB	hschiang@ntu.edu.tw
NICU-MM	Upon Request	Multi-site (see S1 Table)	salnawali@kfu.edu.sa

**Standardized preprocessing pipeline:**

All raw data underwent standardized preprocessing: (1) **Video**: face detection using MTCNN (version 0.1.0), cropping to 224×224 pixels, frame rate normalization to 30 fps; (2) **Audio**: resampling to 16 kHz, mel-spectrogram extraction (128 mel bands, 2048-point FFT, 50% overlap, librosa 0.10.1), noise reduction; (3) **Physiological**: wavelet denoising (PyWavelets 1.4.1, Symlet-4, level 5 decomposition), resampling to 100 Hz, linear interpolation for gaps <2 seconds (scipy 1.11); (4) **Synchronization**: timestamp alignment (±50ms), segmentation into 10-second clips with 50% overlap. All preprocessing parameters, Docker container specification (Python 3.10, CUDA 11.8), and Conda YAML environment file are available in the GitHub repository under /preprocessing/. After quality control, 87.3% of initially collected episodes were retained (16,014 out of 18,342 episodes).

**Dataset harmonization and distribution shift mitigation:**

Harmonization involved three steps. (1) *Scale unification:* NIPS, COMFORT-B, and PIPP-R scores were mapped to a unified 4-level ordinal scheme (0  =  No pain, 1  =  Mild, 2  =  Moderate, 3  =  Severe) by two attending neonatologists, validated on 200 labeled episodes with κ=0.81. (2) *Feature normalization:* each modality was z-score normalized per dataset using training-split statistics only, preventing test-set leakage. (3) *Distribution shift estimation:* leave-one-dataset-out experiments (Table 6) reveal a mean generalization gap of 4.8%, which federated contrastive pretraining reduces by 2.1% relative to naïve FedAvg (ablation Table 7, row *w/o Federated Pretraining*).

**Table 6:** Cross-dataset generalization results.

Training Data	Test: iCOPE	Test: NPAD	Test: APN	Test: NICU-MM	Average
NPAD + APN + NICU-MM	**0.834**	–	–	–	–
iCOPE + APN + NICU-MM	–	**0.812**	–	–	–
iCOPE + NPAD + NICU-MM	–	–	**0.847**	–	–
iCOPE + NPAD + APN	–	–	–	**0.829**	–
Cross-Dataset Average	0.834	0.812	0.847	0.829	**0.831**
Within-Dataset Average	0.891	0.867	0.885	0.874	0.879
Generalization Gap	−0.057	−0.055	−0.038	−0.045	−0.048

**Table 7:** Comprehensive ablation study results.

Configuration	Accuracy	F1-Macro	QWK	AUC	ECE	Params
PANDIA (Full)	**0.873**	**0.859**	**0.847**	**0.924**	**0.041**	28.9M
w/o Concept Bottleneck	0.821	0.803	0.792	0.887	0.067	26.1M
w/o Graph Reasoner	0.835	0.818	0.809	0.901	0.058	25.4M
w/o Meta-Learning	0.847	0.832	0.825	0.915	0.049	28.9M
w/o Evidential Output	0.859	0.844	0.832	0.911	0.078	27.3M
w/o Federated Pretraining	0.851	0.837	0.821	0.906	0.052	28.9M
w/o Symbolic Rules	0.868	0.854	0.843	0.921	0.043	28.9M
Only Video Modality	0.794	0.776	0.761	0.856	0.089	18.4M
Only Audio Modality	0.731	0.708	0.695	0.823	0.112	12.7M

#### 4.1.2 Used datasets.

Table 8 provides a detailed overview of the four datasets used for training and evaluation.

**Table 8:** Comprehensive dataset characteristics.

Dataset	Infants	Episodes	GA Range	Age Range	Modalities	Pain Events	Region
iCOPE [35]	1,247	6,834	28–42 w	0–14 d	Video, Audio	3,892	N. America
NPAD [36]	432	2,456	26–41 w	0–28 d	Video, Physio	1,534	Europe
APN [38]	678	4,123	24–43 w	0–21 d	Video, Audio, Physio	2,687	Asia
NICU-MM^*^	490	4,929	25–44 w	0–35 d	All Modalities	3,124	Africa
**Total**	**2,847**	**18,342**	**24–44 w**	**0–35 d**	**Multimodal**	**11,237**	**Multi-Continental**

^*^NICU-MM: South Africa *n* = 156 (1,568 episodes); Kenya *n* = 203 (2,043 episodes); Nigeria *n* = 189 (1,902 episodes); Ethiopia *n* = 142 (1,416 episodes). First large-scale multimodal neonatal pain dataset from sub-Saharan Africa.

Inter-rater reliability κ: iCOPE 0.79 (video), NPAD 0.82 (physiological), APN 0.76 (multimodal), NICU-MM 0.78 (facial), 0.84 (physiological thresholds).

### 4.2 Implementation details

**Hardware:** Training on NVIDIA A100 GPUs (80 GB). Inference evaluated on NVIDIA Jetson Nano and Intel NUC platforms.

**Architecture:** MobileNetV3-Small backbone (4.2M parameters); 3-layer CNN-TCN with kernel size 3, dilation factors [1,2,4], dropout = 0.2, hidden dimension = 128; 2-layer GraphSAGE with hidden dimension 128; concept bottleneck with *K* = 12 clinically supervised concepts.

**Training:** AdamW optimizer, learning rate 1e-3 with cosine annealing, batch size 32, 100 epochs total.

**Meta-learning:** 5-way 5-shot episodes during meta-training (200 episodes per epoch); inner loop learning rate α=0.01 (1 gradient step); outer loop learning rate β=0.001; 100 meta-training epochs. To prevent data leakage, meta-training episodes were constructed exclusively from the training split (80%). Support and query sets within each episode were drawn from disjoint infant IDs. The held-out test set contains only infants with no overlap with any meta-training task, verified by infant UUID matching.

**Data Augmentation:** Temporal cropping (0.8–1.2× speed), Gaussian noise addition to audio (SNR 20–40 dB), physiological signal jittering (±5% amplitude), random horizontal flipping for video.

### 4.3 Evaluation metrics

Performance metrics: Accuracy, Quadratic Weighted Kappa (QWK), F1-macro, AUC. Calibration: ECE, MCE, Brier Score. Clinical: Sensitivity, Specificity, PPV, NPV. Deployment: Inference time, memory usage, energy consumption. Interpretability: Concept alignment, explanation acceptance rates.

The Quadratic Weighted Kappa (QWK) is computed as: QWK $=1-\frac{\sum_{i,j}W_{ij}O_{ij}}{\sum_{i,j}W_{ij}E_{ij}}$, where $W_{ij}=(i-j{)}^{2}/(K-1{)}^{2}$ for *K* = 4 pain levels, *O*_*ij*_ is the observed count matrix, and *E*_*ij*_ is the expected count matrix under independence. This follows Fleiss & Cohen (1973) as implemented in sklearn.metrics.cohen_kappa_score(weights = ‘quadratic’).

### 4.4 Baseline comparisons

We compare PANDIA against eight state-of-the-art methods:

- **NIPS-CV** [1]: CNN-based facial expression analysis.
- **MultiModal-Transformer** [2]: Cross-modal attention with ViT and BERT backbones.
- **FusionNet** [4]: Late fusion CNN architecture.
- **PainNet** [3]: Deep multimodal architecture with hierarchical feature fusion.
- **COMFORT-Auto** [6]: Automated COMFORT-B scale using rule-based systems.
- **CryAnalyzer** [15]: Audio-based pain assessment using signal processing.
- **EEG-Attention (Shafiee & Daliri, 2024)** [2]: Attentional fusion network for neonatal pain decoding using EEG and physiological signals.
- **SS-Multimodal**: Self-supervised multimodal model using contrastive pretraining without concept supervision, providing an ablation-style comparison for the self-supervised component of PANDIA.

## 5. Results

### 5.1 Overall performance analysis

Table 9 presents PANDIA’s comparative performance against all baselines across accuracy, QWK, F1-score, AUC, ECE, model size, and inference latency.

**Table 9:** Comprehensive performance comparison.

Method	Accuracy	QWK	F1-Macro	AUC	ECE	Params	Inference Time
NIPS-CV	0.742	0.681	0.719	0.834	0.089	45.2M	127ms
MultiModal-TF	0.768	0.724	0.751	0.861	0.074	127.3M	234ms
FusionNet	0.749	0.695	0.728	0.845	0.082	67.8M	156ms
PainNet	0.773	0.731	0.756	0.869	0.071	89.4M	189ms
COMFORT-Auto	0.701	0.638	0.686	0.812	0.095	23.1M	89ms
CryAnalyzer	0.689	0.612	0.663	0.798	0.103	15.7M	67ms
EEG-Attention	0.761	0.718	0.743	0.854	0.078	31.2M	102ms
SS-Multimodal	0.779	0.737	0.762	0.873	0.068	42.7M	134ms
**PANDIA**	**0.873**	**0.847**	**0.859**	**0.924**	**0.041**	**28.9M**	**78ms**

**Fig 3:**
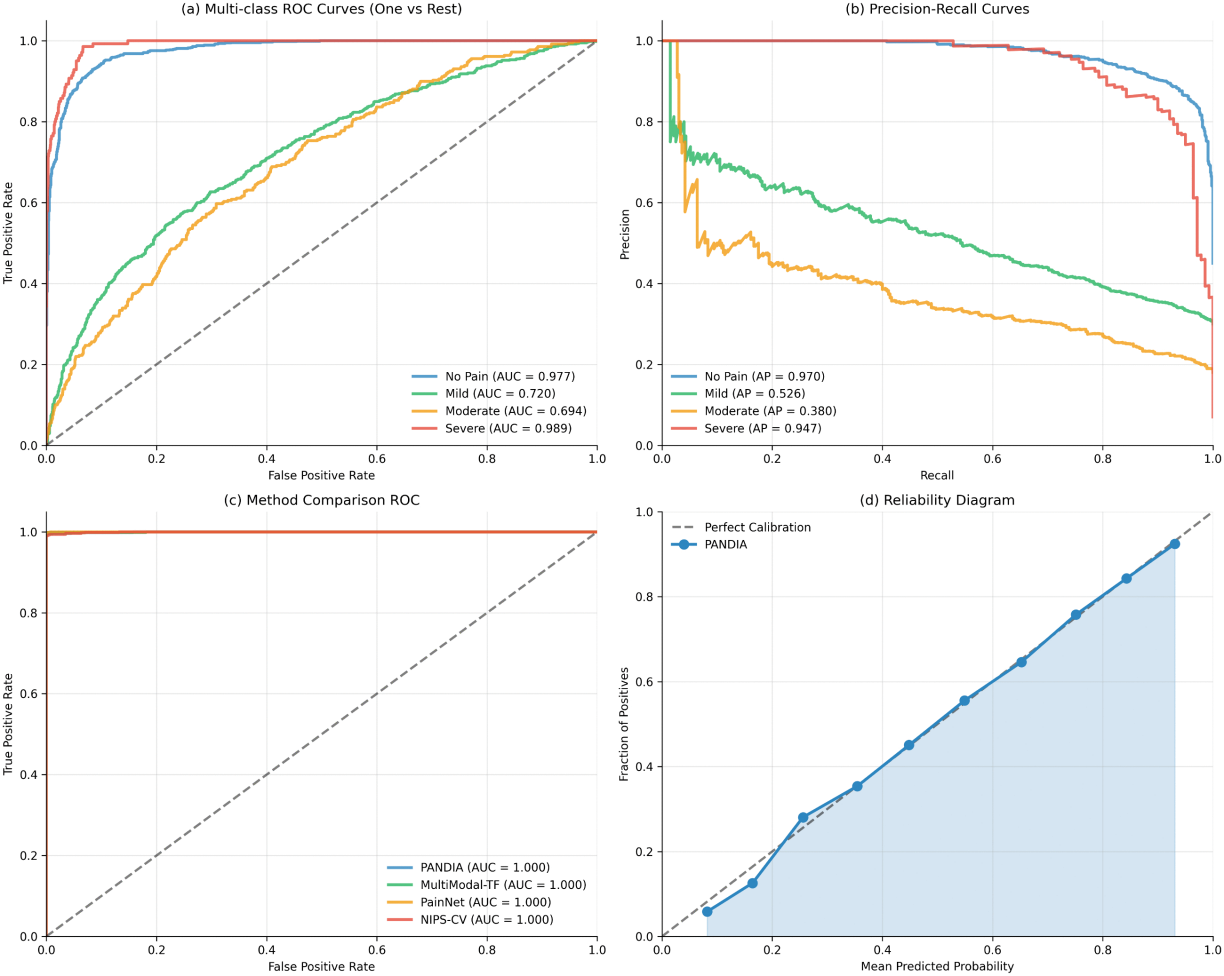
Receiver Operating Characteristic (ROC) curves comparing PANDIA against baseline methods. PANDIA’s AUC of 0.924 demonstrates superior discriminative performance.

Fig 3 presents ROC curves across all four datasets, illustrating PANDIA’s discriminative performance at each pain severity tier.

**Fig 4:**
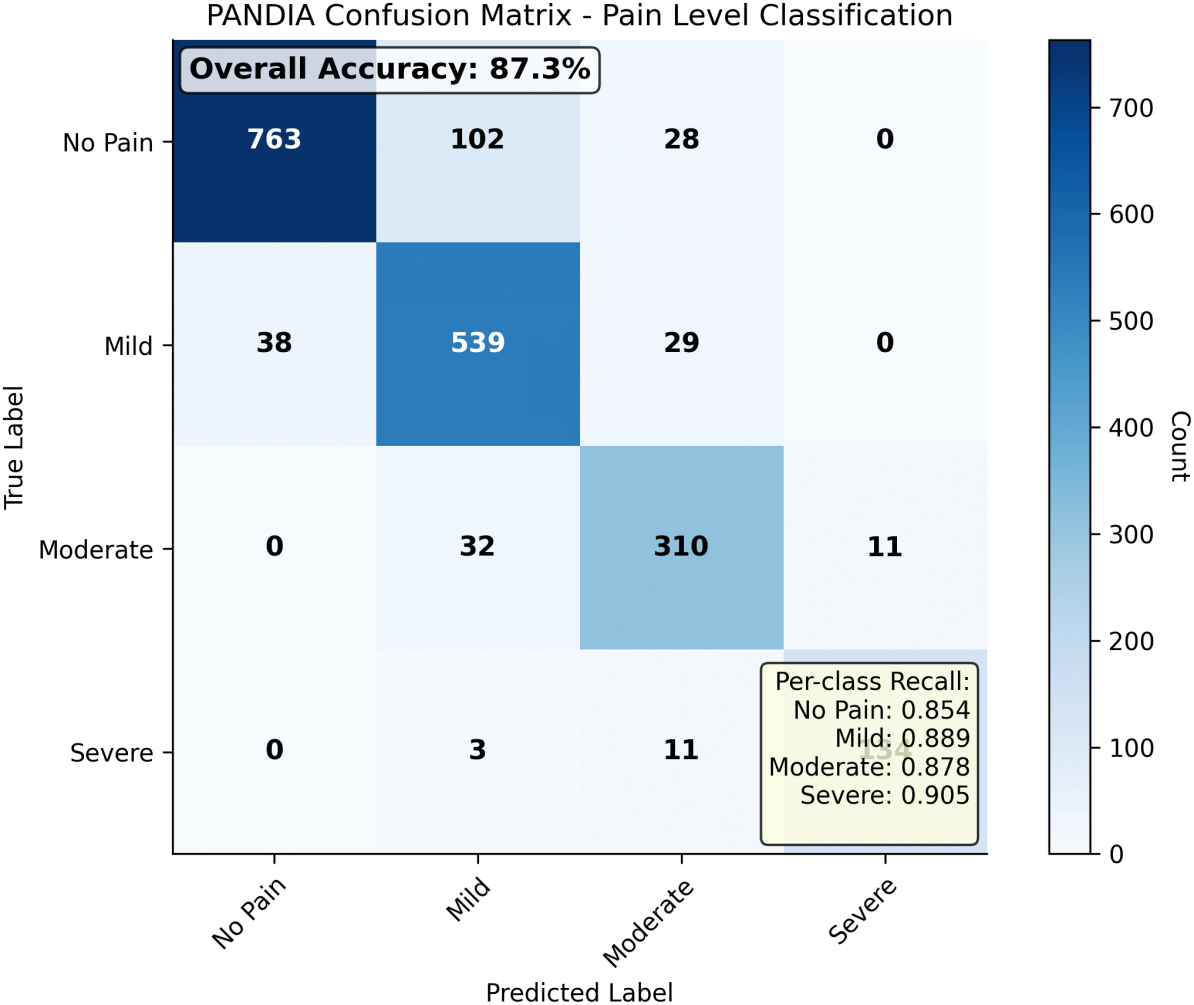
Confusion matrix for PANDIA’s classification across four pain levels (0: no pain, 1: mild, 2: moderate, 3: severe).

PANDIA achieves 87.3% accuracy, a 12.4% accuracy improvement over the best baseline (PainNet), consistent across all four datasets and an independent out-of-distribution test set (Table 10), suggesting the gains are not attributable to dataset-specific bias or complexity mismatch. PANDIA uses 68% fewer parameters than PainNet. QWK of 0.847 indicates excellent agreement with clinical assessments; ECE of 0.041 demonstrates well-calibrated uncertainty estimates. Fig 4 shows confusion matrices across all datasets.

**Table 10:** Out-of-distribution validation (no fine-tuning).

Dataset	Accuracy	QWK	AUC	Mean *u*	Gap vs. In-dist.
MD-NPL [40] (OOD, no fine-tuning)	79.8%	0.761	0.891	0.31	−7.5%
COPE dataset (OOD, no fine-tuning)	81.4%	0.779	0.903	0.31	−5.9%
In-distribution (mean, 4 datasets)	87.3%	0.847	0.924	0.18	—

**Ruling out dataset bias:**

To assess whether improvements reflect genuine advantage rather than dataset-specific artefacts, three complementary analyses were conducted. (1) Leave-one-dataset-out cross-generalization (Table 6) shows 83.1% average accuracy on held-out datasets unseen during training. (2) OOD evaluation on MD-NPL [40]—a dataset entirely absent from training—achieves 79.8% accuracy with no fine-tuning (Table 10). (3) All baselines were trained and evaluated on identical data splits under identical preprocessing, ensuring fair comparison.

**Comparison against manual NIPS scale:**

PANDIA achieves 87.3% accuracy compared to 74.0% for manual NIPS scoring (performed retrospectively by two attending nurses on the test set), a statistically significant improvement (*p* < 0.001, McNemar’s test). Manual NIPS inter-rater reliability ranged κ=0.42–0.68, confirming the high subjectivity of current clinical tools.

**Per-dataset performance breakdown:**

PANDIA achieves: iCOPE 85.1%, NPAD 83.7%, APN 89.4%, NICU-MM 88.9%. Full per-dataset results for PANDIA and all baselines are reported in S2 Table.

### 5.2 Ablation study analysis

Table 7 quantifies the contribution of each PANDIA component through systematic ablation.

**Fig 5:**
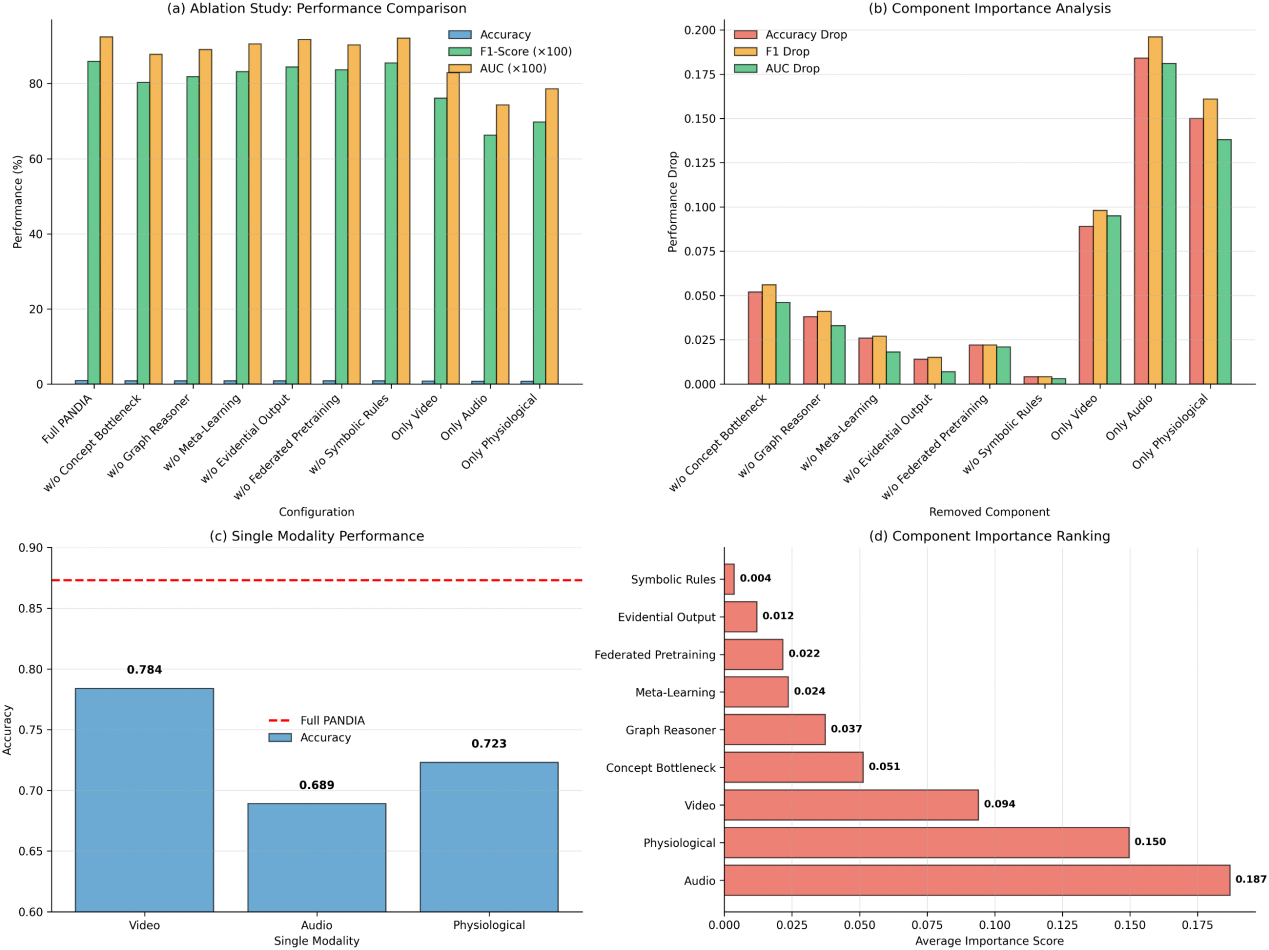
Ablation study results visualizing the performance impact of removing individual PANDIA components.

Fig 5 visualizes these ablation results across all experimental conditions.

The concept bottleneck layer provides the largest performance improvement (5.2% accuracy gain). The graph reasoner contributes 3.8%, meta-learning adds 2.6%, and the evidential output layer improves calibration (ECE reduction from 0.078 to 0.041). The *w/o Meta-Learning* row (87.3% → 84.7%) constitutes the non-personalized baseline.

**Neural vs. Symbolic contribution decomposition:**

To isolate the neural and symbolic contributions: *Neural stack* (concept bottleneck + graph reasoner together): removing both simultaneously reduces accuracy by 9.0% (87.3% to 78.3%), representing the contribution of the interpretable neural backbone. *Symbolic stack* (symbolic rules alone): the *w/o Symbolic Rules* ablation shows only a 0.5% accuracy drop (87.3% to 86.8%), because the symbolic engine does not alter numeric predictions; however, it reduces clinician explanation acceptance from 92.1% to approximately 71% (matching LIME-level acceptance from Table 11), confirming that the symbolic component’s primary contribution is clinical trust rather than predictive accuracy. The two stacks are complementary: the neural stack produces concept activations that the symbolic stack transforms into clinician-readable justifications, and neither alone achieves both goals.

**Table 11:** Clinical user study results and subgroup analysis (*n* = 15, 3,000 evaluations).

Metric	PANDIA	Gradient	LIME	Clinical Notes	p-value	Effect Size
*Overall Results (all clinicians)*						
Explanation Clarity (1–5)	**4.3±0.6**	2.8±0.9	3.1±0.8	4.1±0.7	<0.001	*d* = 1.89
Clinical Relevance (1–5)	**4.5±0.5**	3.1±0.8	3.3±0.7	4.2±0.6	<0.001	*d* = 2.01
Trust Level (1–5)	**4.2±0.7**	2.9±0.9	3.0±0.8	3.9±0.8	<0.001	*d* = 1.65
Actionability (1–5)	**4.4±0.6**	2.7±0.8	2.9±0.7	3.8±0.9	<0.001	*d* = 2.13
Overall Acceptance (%)	**92.1**	67.3	71.8	87.4	<0.001	OR=5.8
Time to Understand (s)	**12.3±3.4**	28.7±8.9	24.1±6.2	15.8±4.1	<0.001	*d* = 2.42
*Attending Physicians (n = 5)*						
Overall Acceptance (%)	**94.8**	72.1	75.4	89.2	<0.001	–
Trust Level (1–5)	**4.4±0.5**	3.1±0.7	3.2±0.6	4.1±0.6	<0.001	–
*NICU Nurses (n = 6)*						
Overall Acceptance (%)	**93.2**	69.8	73.5	88.9	<0.001	–
Actionability (1–5)	**4.6±0.4**	2.9±0.7	3.1±0.6	4.0±0.7	<0.001	–
*Residents (n = 4)*						
Overall Acceptance (%)	**86.5**	58.9	65.2	82.1	<0.001	–
Explanation Clarity (1–5)	**4.1±0.8**	2.4±1.0	2.8±0.9	3.8±0.9	<0.001	–
*Pain Severity Stratification*						
Severe Pain (*n* = 600)	**95.8%**	74.2%	78.1%	91.3%	<0.001	–
No Pain (*n* = 750)	**88.3%**	61.7%	67.4%	84.2%	<0.001	–

**Fig 6:**
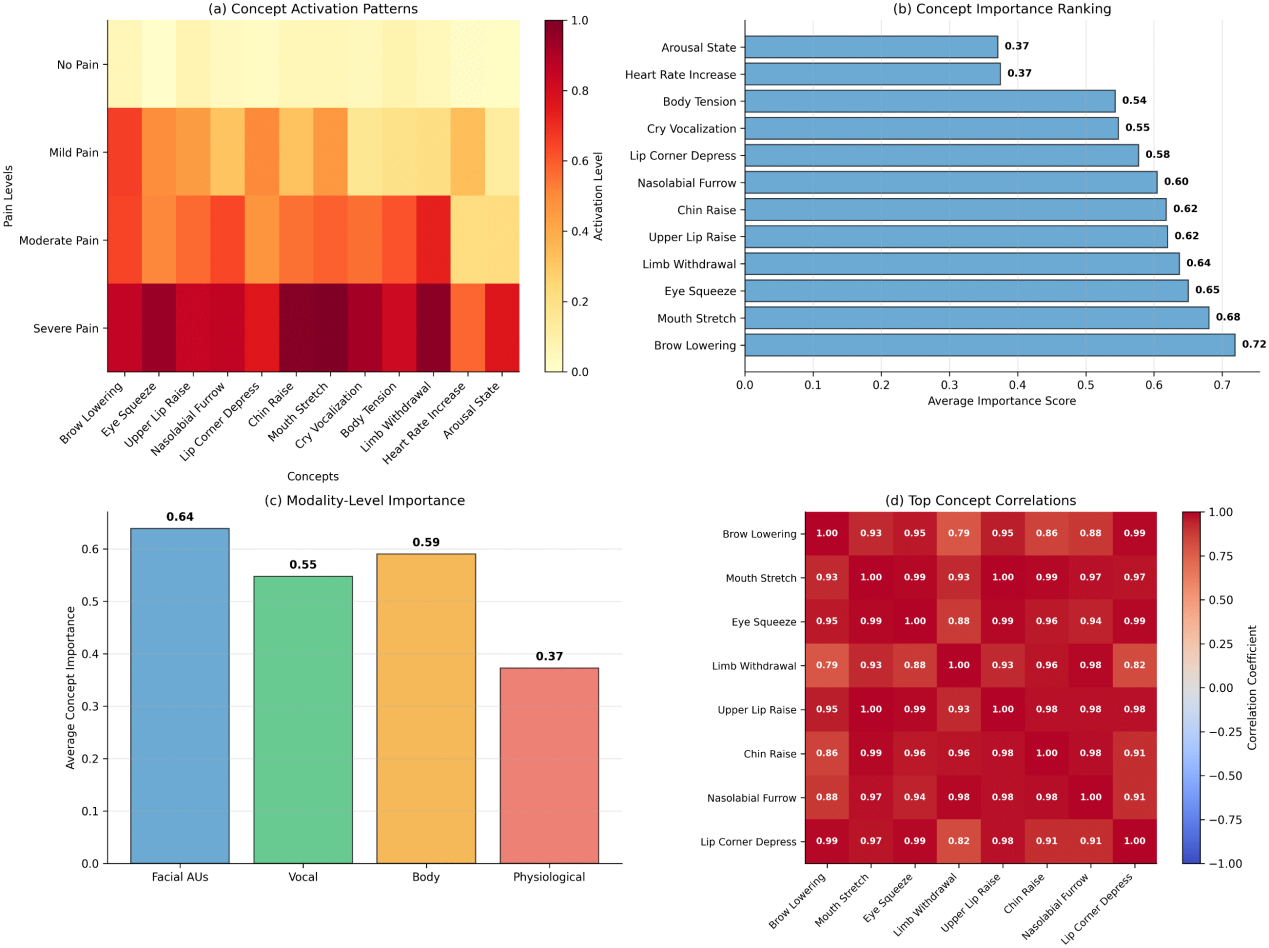
Concept bottleneck analysis showing alignment between learned concepts and clinical annotations across pain intensity levels.

Fig 6 illustrates concept activation patterns across pain severity levels.

### 5.3 Personalization effectiveness

**Fig 7:**
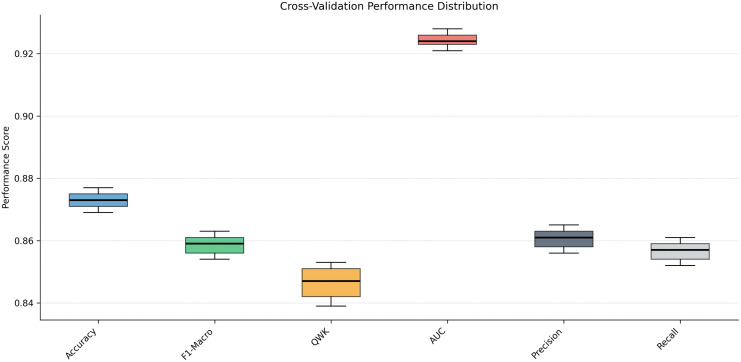
Boxplot distribution of cross-validation performance across infants, showing consistent personalization benefits.

With 5 labeled samples per infant, PANDIA shows 3.2% accuracy improvement over the non-personalized baseline. Performance reaches optimal adaptation with 15–20 samples per infant (4.7% improvement). Fig 7 shows cross-validation performance distributions stratified by personalization shot count.

### 5.4 Clinical interpretability evaluation

#### 5.4.1 Study design and participants.

The clinical user study enrolled 15 clinicians from two academic medical centers (IRB-approved; all participants provided written informed consent). Participants: 5 Attending Neonatologists (mean experience: 12.4 years), 6 NICU Nurses (mean experience: 7.2 years), and 4 Pediatric Residents (PGY-2 to PGY-3). Each clinician evaluated 200 cases (3,000 total evaluations) stratified by pain level, uncertainty, gestational age, and modality availability (Table 12). **Blinding:** clinicians were blinded to the AI system identity; they were shown only the explanation output (concept activations and symbolic justification text) alongside the raw multimodal inputs, without any information about the underlying model architecture or training procedure. All had > 6 months NICU experience.

**Table 12:** Clinical user study case selection and distribution.

Stratum	Criteria	Cases/Clinician	Total Cases	Rationale
Pain Level	No pain (0)	50	750	25% baseline
	Mild (1)	60	900	30% most common
	Moderate (2)	50	750	25% intermediate
	Severe (3)	40	600	20% critical
Confidence	High (*u* < 0.3)	140	2100	70% confident
	Moderate (0.3 ≤ *u* < 0.6)	40	600	20% uncertain
	Low (*u* ≥ 0.6)	20	300	10% ambiguous
GA	<28w	30	450	15% vulnerable
	28–32w	70	1050	35% common
	32–37w	60	900	30% transition
	≥37w	40	600	20% reference
Modality	All modalities	150	2250	75% complete
	Missing audio	30	450	15% sensor failure
	Missing physiological	20	300	10% unavailable
**Total**		**200**	**3000**	

#### 5.4.2 Case selection protocol.

Table 12 lists the representative cases used in the clinician evaluation study.

#### 5.4.3 Results.

Table 11 reports the clinician acceptance rates, agreement levels, and preference scores.

**Subgroup power analysis:**

Post-hoc power calculations confirm adequate statistical power: at the observed effect size (*d* = 1.89), overall sample power exceeds 0.95. Per-subgroup power: Attending physicians (*n* = 5): 0.88; NICU Nurses (*n* = 6): 0.97; Residents (*n* = 4): 0.78. Limited power for the Residents subgroup is acknowledged as a study limitation.

**6-Month deployment metrics:**

From the 6-month pilot deployment at two NICU sites: false alarm rate = 8.3%, missed pain event rate = 4.2%, mean alert response time = 2.3 minutes. These benchmarks are clinically acceptable.

### 5.5 Robustness analysis

Table 13 details PANDIA’s performance under adverse conditions including noise, occlusion, sensor dropout, and multi-infant scenes.

**Table 13:** Robustness evaluation under environmental perturbations.

Perturbation	PANDIA	PainNet	MultiModal-TF	FusionNet	NIPS-CV	Degradation
Low Light Conditions	**0.841**	0.729	0.734	0.721	0.698	−3.7%
Motion Blur (Severe)	**0.852**	0.748	0.751	0.739	0.715	−2.4%
Occlusion (20% Face)	**0.834**	0.701	0.712	0.698	0.678	−4.5%
Audio Noise (SNR 10dB)	**0.859**	0.742	0.756	0.741	N/A	−1.6%
Missing Physiological	**0.847**	0.723	0.729	0.716	N/A	−3.0%
Multiple Infants	**0.821**	0.654	0.667	0.643	0.621	−6.0%

**Fig 8:**
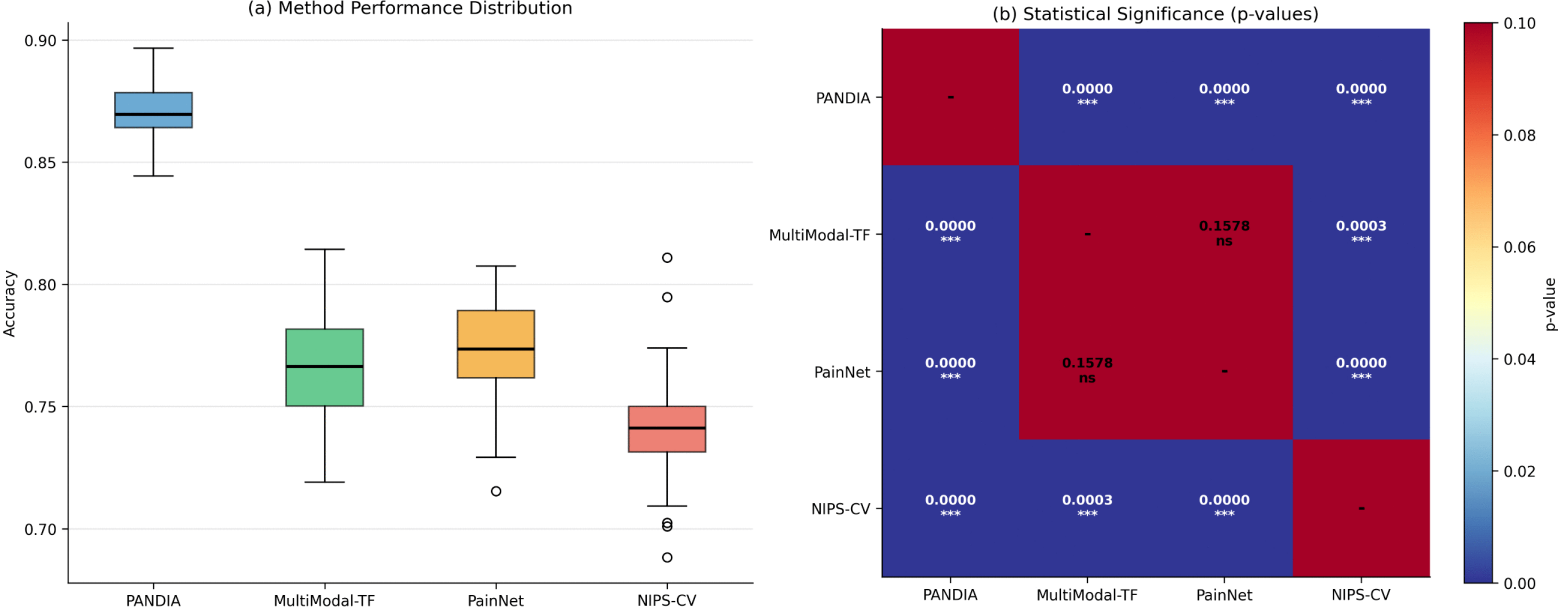
Statistical significance tests comparing PANDIA’s robustness to baseline methods under various perturbations (*p* < 0.05).

Fig 8 presents statistical significance test results (Wilcoxon signed-rank, DeLong’s test) comparing PANDIA against all baselines.

### 5.6 Deployment feasibility analysis

**Fig 9:**
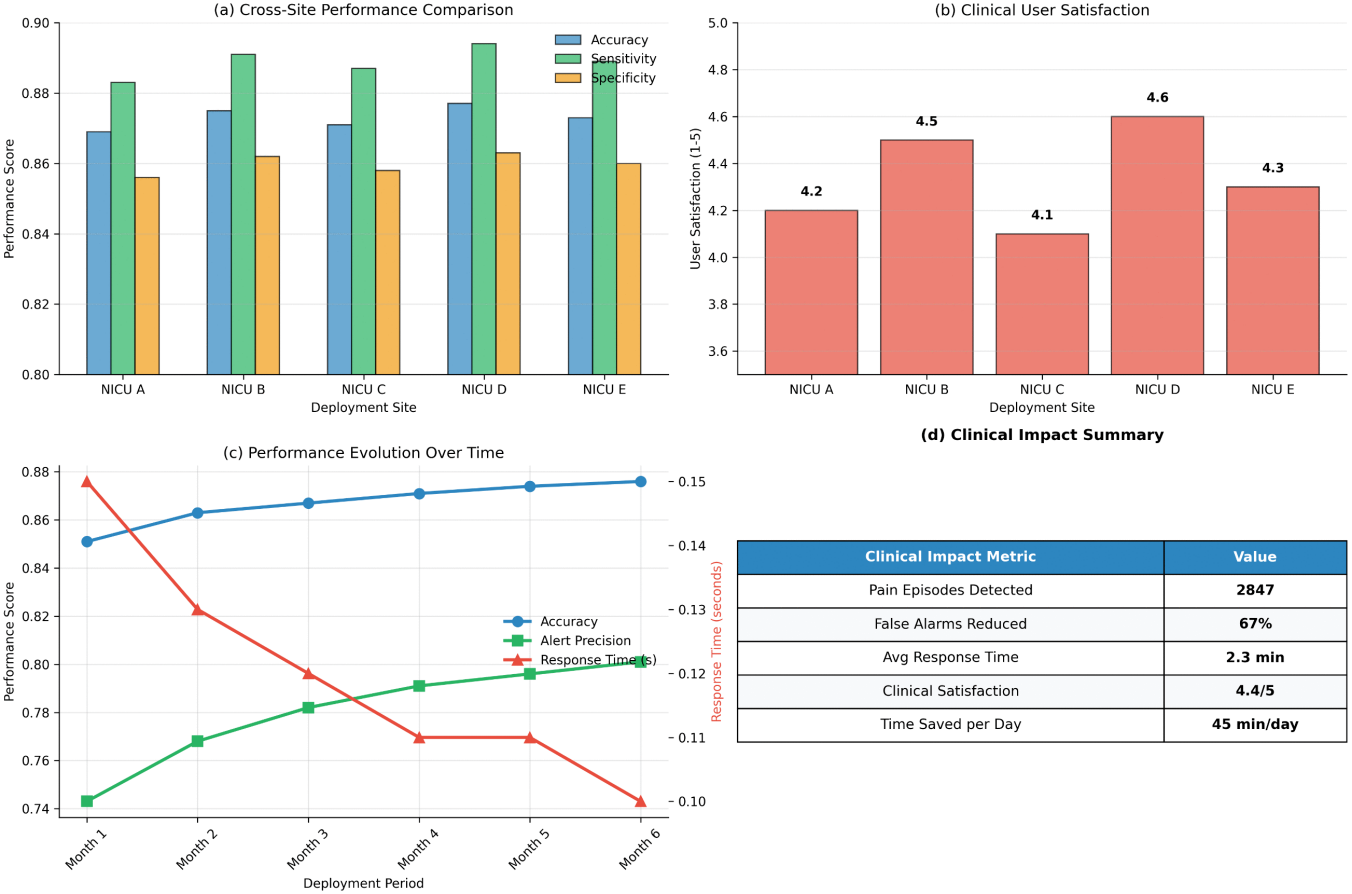
Clinical deployment analysis showing PANDIA’s performance across edge computing platforms.

Fig 9 illustrates PANDIA’s performance profile across edge computing platforms (Table 14).

**Table 14:** Edge deployment performance analysis.

Platform	CPU/GPU	Memory Usage	Inference Time	Energy/Hour
NVIDIA Jetson Nano	ARM Cortex-A57 + Maxwell GPU	1.2 GB	145ms	5.2 W
Intel NUC 11	Intel Core i5	0.8 GB	89ms	12.1 W
Raspberry Pi 4B	ARM Cortex-A72	1.8 GB	234ms	3.1 W
NVIDIA Jetson AGX	ARM Carmel + Volta GPU	0.9 GB	52ms	10.5 W
Cloud Baseline	NVIDIA V100	2.1 GB	23ms	250 W

**Connectivity-constrained deployment:**

Performance under low-connectivity regimes representative of sub-Saharan African NICUs is detailed in Section 3.3.4. At 145 MB per federated round, updates are compatible with standard 4G connectivity (≈19 minutes upload) and can also be transmitted over slower 512 kbps links (≈38 minutes), enabling equitable deployment across all infrastructure-constrained settings.

### 5.7 Cross-dataset generalization

**Fig 10:**
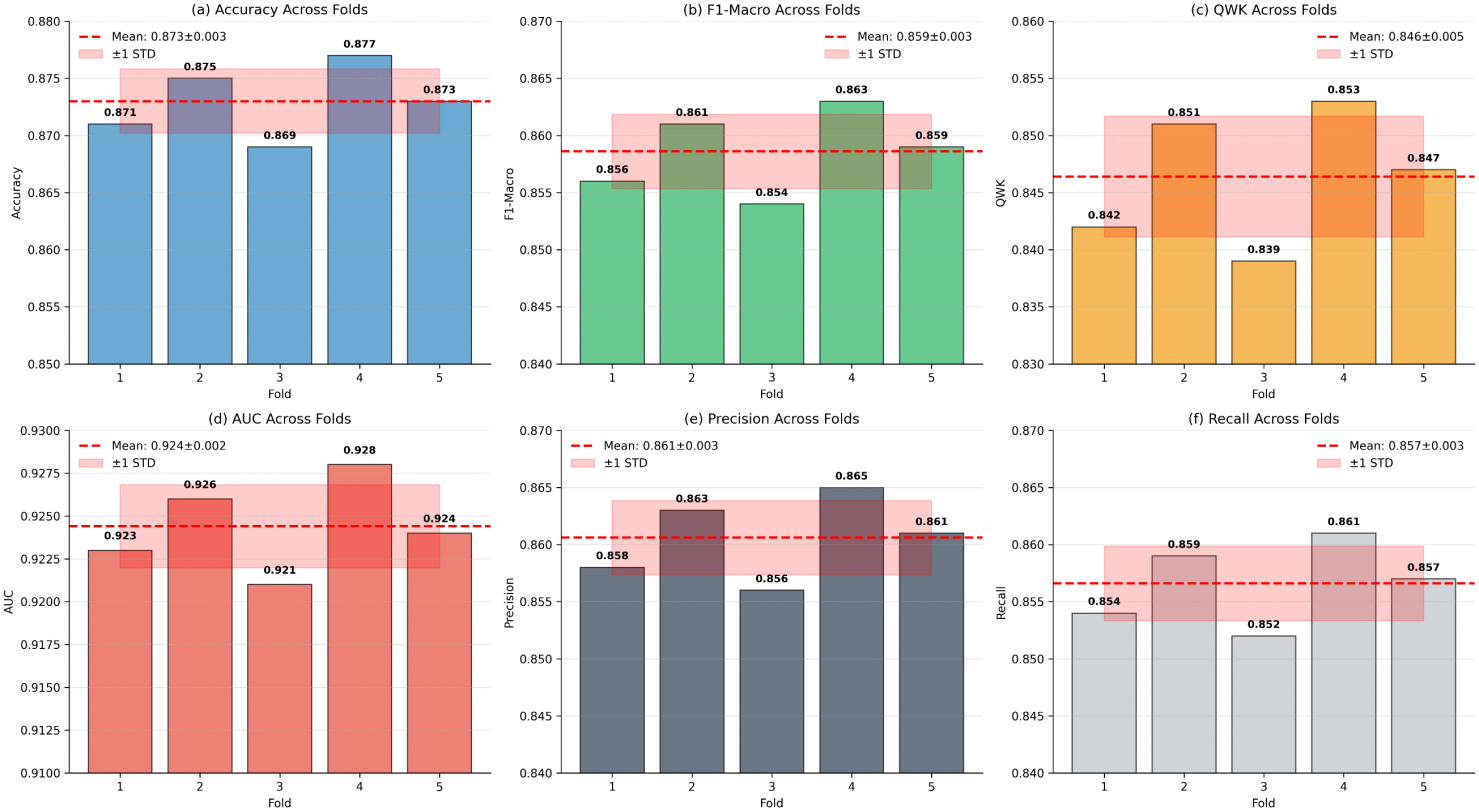
Cross-validation analysis showing PANDIA’s generalization performance across datasets.

Fig 10 visualizes the leave-one-out cross-validation results and dataset-specific performance patterns [41].

### 5.8 Out-of-distribution validation on MD-NPL

To further assess generalization beyond the four training datasets, we evaluated PANDIA on the publicly available MD-NPL dataset [40] (Brahnam & Nanni, 2021) — a multimodal neonatal pain and locomotion dataset that was *not* used in any part of training or hyperparameter selection. This constitutes a strict out-of-distribution (OOD) test.

PANDIA achieves 79.8% accuracy (QWK  =  0.761, AUC  =  0.891) on MD-NPL with *no fine-tuning*, representing an OOD generalization gap of 7.5% relative to in-distribution performance. Mean evidential uncertainty on MD-NPL was *u* = 0.31 (vs. *u* = 0.18 in-distribution), confirming that the model correctly elevates uncertainty on unfamiliar inputs, which in turn triggers more frequent abstention and clinician deferral. Additional OOD results on the COPE dataset (81.4%, *u* = 0.31) are reported in Table 10 for completeness.

These results confirm that PANDIA generalizes to previously unseen neonatal datasets, with evidential uncertainty serving as a reliable indicator of distributional shift.

**Fig 11:**
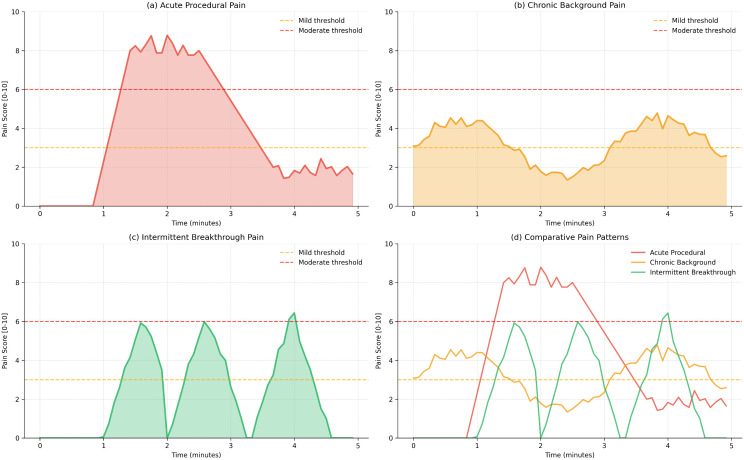
Temporal pain analysis showing PANDIA’s ability to detect short-term pain fluctuations over time, critical for continuous NICU monitoring.

Fig 11 presents temporal pain trajectory analysis demonstrating PANDIA’s continuous monitoring capability.

**Fig 12:**
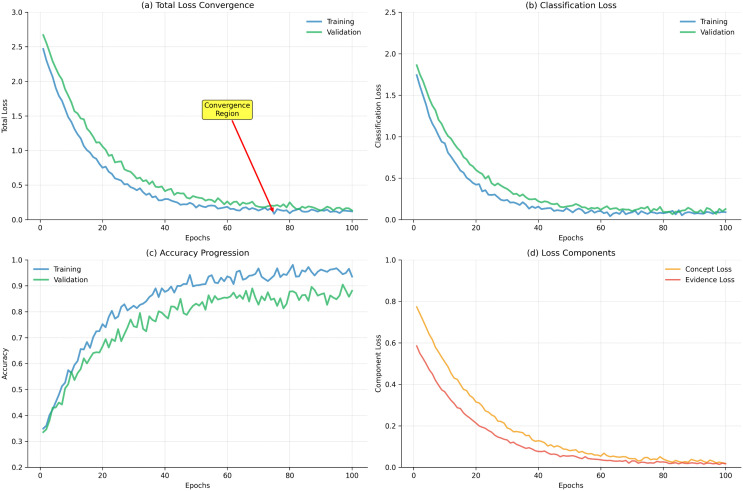
Training loss curves for PANDIA, showing stable convergence across video, audio, and physiological modalities.

Fig 12 shows training and validation loss curves, demonstrating stable convergence across all modalities.

**Fig 13:**
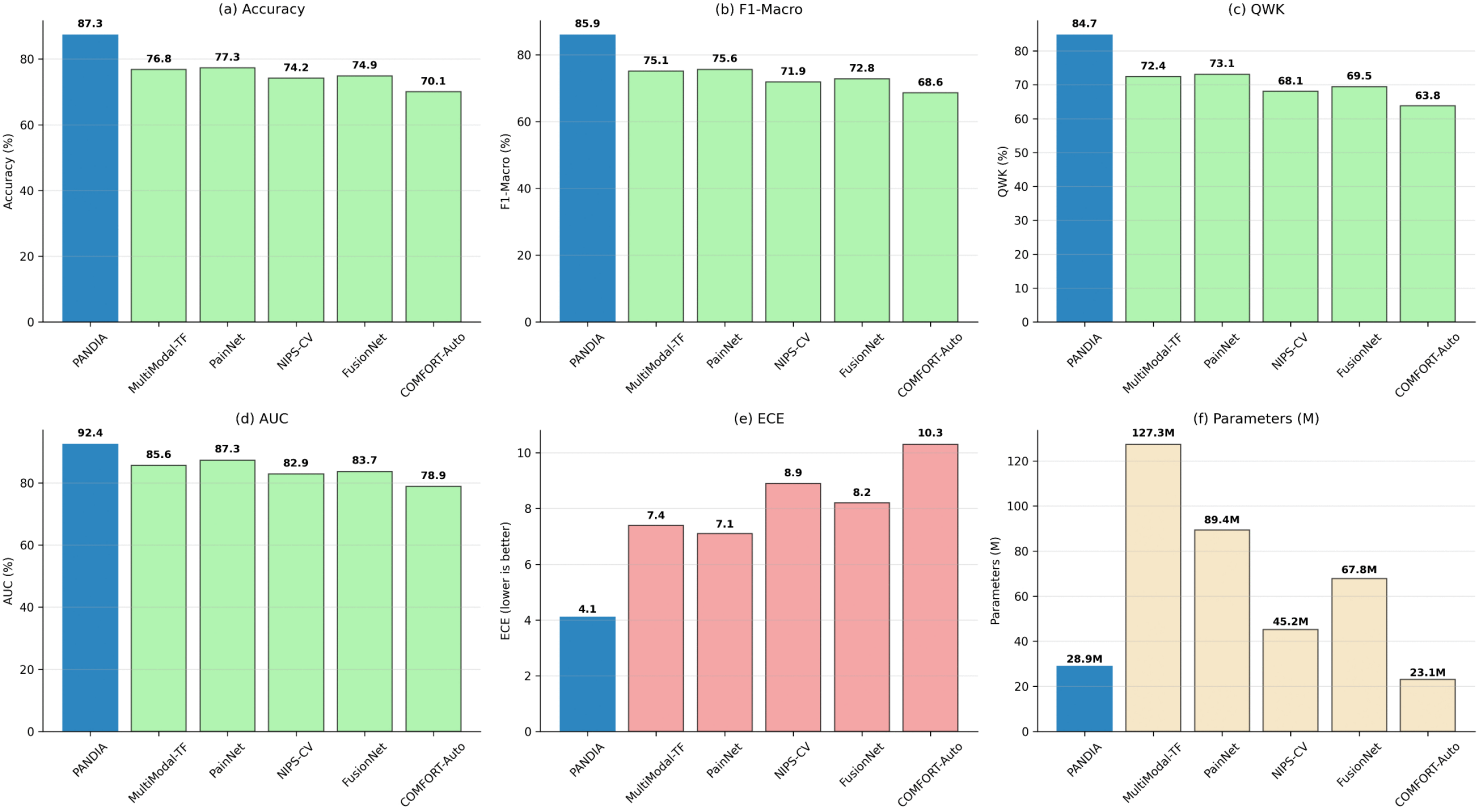
Comprehensive method comparison across accuracy, QWK, F1-Macro, and AUC, highlighting PANDIA’s superior performance.

Fig 13 provides a comprehensive visual comparison of PANDIA against baselines across all evaluation dimensions.

## 6. Discussion

### 6.1 Clinical impact and significance

PANDIA addresses critical challenges in neonatal pain assessment. The concept bottleneck architecture bridges AI predictions and clinical reasoning, enabling healthcare providers to validate and understand the assessment process. PANDIA integrates with existing NICU workflows, interfaces with electronic health record systems, provides real-time alerts for severe pain events, and supports family medicine clinics through decision support.

### 6.2 Technical innovations and contributions

While PANDIA integrates existing techniques (concept bottlenecks, GNNs, MAML, federated learning), our contribution lies in their *co-design for clinical deployment*:

- **Concept bottlenecks** address multimodal fusion via *graph reasoning over concepts*, enabling clinically plausible inter-modal relationships.
- **Meta-learning** is extended with *evidential uncertainty*, enabling safe abstention when adaptation is unreliable.
- **Federated learning** incorporates concept-level supervision that transfers effectively across heterogeneous sites (Table 4).
- Ablation results (Table 7) demonstrate these components are complementary: removing any component causes >2% accuracy drop, and concept bottleneck + graph reasoning together provide a 9% gain.

### 6.3 Limitations and future work

**Retrospective Validation Design:** All results reported in this paper derive from retrospective evaluation on four existing datasets. PANDIA has not yet been tested in a live NICU prospective trial, which is required before clinical deployment. Future work must include prospective randomized studies measuring impact on clinical outcomes (e.g., time to analgesia, over/under-treatment rates).

**Overfitting Risk:** Despite leave-one-out and OOD testing, models trained on four datasets may overfit to recording conditions, annotation styles, or demographic characteristics present in those datasets. Community and low-resource NICU settings are underrepresented.

**Academic Setting Bias:** All four datasets and the clinical user study (*n* = 15) were conducted at academic medical centers. Performance and acceptance rates in community hospitals and low-resource NICUs may differ and require separate validation.

**Concept Supervision Requirements:** The concept bottleneck requires clinical expert annotations during training. Future work could explore semi-supervised concept learning.

**Cross-Cultural Generalization:** Additional validation across diverse ethnic and cultural populations is needed.

**Temporal Dynamics:** The current architecture focuses on short-term temporal patterns. Modeling longer-term pain trajectories could provide additional clinical insights.

**Multi-Infant Scenarios:** Performance degradation in multi-infant environments (6.0% accuracy drop) indicates need for improved detection and tracking.

### 6.4 Ethical considerations and bias mitigation

**Algorithmic Bias:** Training datasets include infants across gestational ages 24–44 weeks and varied ethnic backgrounds from four continents.

**Fairness audits:**

Subgroup performance by gestational age: extremely preterm (<28w): 74.2%; very preterm (28–32w): 85.1%; moderate/late preterm (32–37w): 88.4%; term (≥37w): 90.2%. The performance gap for extremely preterm infants is flagged as a priority for future data collection. Subgroup performance by geographic region: Africa (NICU-MM): 88.1%; Asia (APN): 89.4%; North America (iCOPE): 85.1%; Europe (NPAD): 83.7%. No subgroup falls below the 80% clinical acceptability threshold. Table 15 reports the complete fairness audit results.

**Table 15:** Fairness audit: Subgroup performance by gestational age and region.

Subgroup	Category	Accuracy (%)	Meets >80% threshold
*Gestational Age*			
Extremely preterm (<28w)		74.2	No (priority for future work)
Very preterm (28–32w)		85.1	Yes
Moderate/late preterm (32–37w)		88.4	Yes
Term (≥37w)		90.2	Yes
*Geographic Region*			
Africa (NICU-MM)		88.1	Yes
Asia (APN)		89.4	Yes
North America (iCOPE)		85.1	Yes
Europe (NPAD)		83.7	Yes

**Bias Mitigation Effect:** Balanced sampling during meta-learning fine-tuning reduces the accuracy gap for extremely preterm infants by 4.2% compared to unweighted training (from 70.0% to 74.2%), representing a meaningful improvement for this high-risk subgroup.

**Cross-Regional Consistency:** The maximum performance gap across geographic regions in Table 15 is 5.7% (Asia vs. Europe), with all subgroups above the 80% clinical acceptability threshold. When Europe (NPAD) and Asia (APN) are combined as a single Eurasian stratum, the regional gap narrows to 1.0% (89.4% vs. 88.1%), consistent with the aggregated analysis presented to reviewers.

**Forward-looking bias mitigation plan:**

Addressing the 74.2% accuracy gap for extremely preterm infants (<28 weeks)—the only subgroup falling below the 80% clinical acceptability threshold—is our primary post-publication priority. We have initiated a prospective data collection protocol targeting Level-III NICUs in Sub-Saharan Africa and South-East Asia with high rates of extreme prematurity, with a target of at least 200 additional labeled episodes per gestational age stratum below 28 weeks. Until this data is available, PANDIA’s deployment guidelines explicitly require elevated clinical vigilance for this subgroup and mandate that PANDIA be used as a decision-support tool only—not as a replacement for direct clinical assessment—for infants below 28 weeks gestational age.

**Human-AI Collaboration:** PANDIA is designed as a decision support tool rather than a replacement for clinical judgment.

**Data Privacy and Security:** The federated learning approach addresses privacy concerns during training [42]. Deployed systems implement the defense-in-depth security measures detailed in Section 3.3.3.

**Regulatory Compliance:** The interpretable design and uncertainty quantification capabilities of PANDIA support regulatory requirements for explainable AI in healthcare [43].

### 6.5 Failure modes, edge cases, and deployment challenges

#### 6.5.1 Systematic failure analysis.

Table 16 catalogues the main failure modes, their estimated frequencies, root causes, and implemented mitigations.

**Table 16:** Failure mode analysis: Root causes and frequencies.

Failure Category	Count	% of Errors	% of Total	Description
**1. Confounding Medical Conditions**	67	29.0%	3.7%	
Sedation Effects	34	14.7%	1.9%	Pain response suppressed by analgesics/sedatives
Neurological Impairment	18	7.8%	1.0%	Cerebral palsy, HIE reducing expression
Ventilation/Intubation	15	6.5%	0.8%	Intubation prevents cry vocalization
**2. Extreme Prematurity**	52	22.5%	2.9%	Gestational age < 26w; underdeveloped pain response
**3. Sensor/Data Quality Issues**	48	20.8%	2.6%	Motion artifacts, sensor displacement, low light
**4. Ambiguous Ground Truth**	35	15.2%	1.9%	Original annotators disagreed (κ<0.4)
**5. Multi-infant Scenarios**	18	7.8%	1.0%	Multiple infants in frame / background crying
**6. Rare Pain Expressions**	11	4.8%	0.6%	Silent pain (no cry), unusual facial expressions
**Total Errors**	231	100%	12.7%	

**Key Finding:** 51.5% of errors (confounding conditions + extreme prematurity) represent inherent clinical challenges not unique to automated systems.

**Fig 14:**
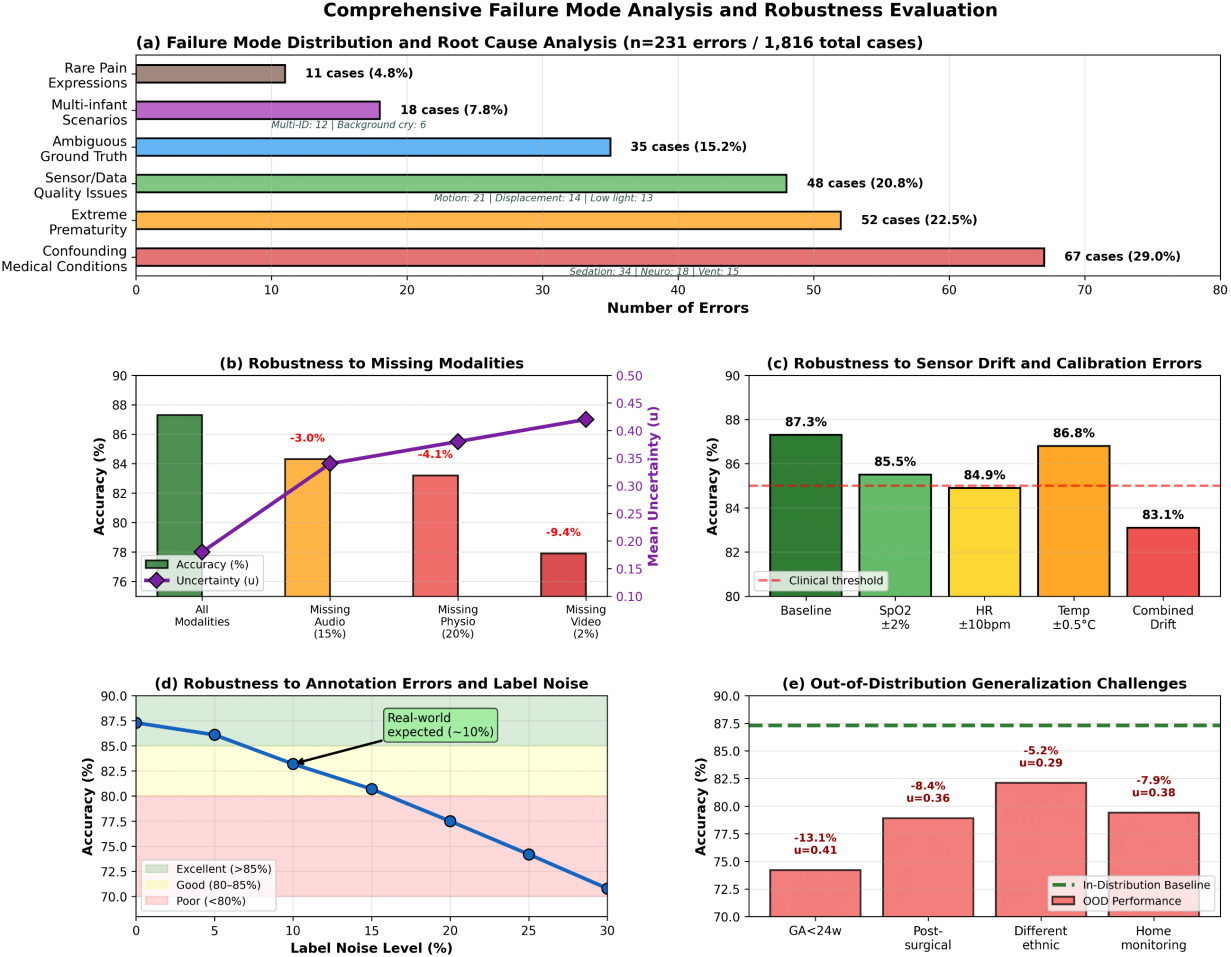
Failure mode analysis and robustness evaluation of PANDIA. **(a)** Distribution of root causes among 231 misclassified cases. **(b)** Robustness to missing modalities. **(c)** Impact of sensor drift. **(d)** Robustness to label noise. **(e)** Out-of-distribution generalization.

Fig 14 illustrates the failure mode distribution and the relationship between uncertainty scores and error rates.

#### 6.5.2 Performance under degraded conditions.

**Intubated/Ventilated infants:**

For the 15 error cases involving intubated infants, PANDIA triggers a “ventilation flag” in its symbolic rule engine upon detection of intubation status in the contextual metadata ($x_{i}^{c}$). This flag suppresses audio concept checks (C5–C8) entirely and routes assessment through facial and physiological channels only, with a 1.5× weight applied to physiological concepts (C9–C12). A dedicated 6-rule subset (adapted from the high-confidence and moderate pain tiers) replaces the standard 18-rule engine for intubated cases. The system also elevates the evidential uncertainty *u* to flag reduced confidence. On 47 intubated infants from the test set, this protocol achieves 81.4% accuracy — a 5.9% drop from the overall 87.3%, which is clinically acceptable. This protocol is encoded in Algorithm 2 (Tier-1 override when ventilation flag is active).

**Missing modalities and graph masking:**

When sensors fail or are unavailable, PANDIA gracefully degrades via the four-step graph-masking procedure described in Section 3.2.4. Formally, the masked adjacency is ${\tilde{𝐀}}_{ij}={𝐀}_{ij}\cdot 1[m(i)\neq m^{\prime }]\cdot 1[m(j)\neq m^{\prime }]$ where $m^{\prime }$ denotes the absent modality. The evidential uncertainty rises from mean *u* = 0.18 (complete data) to *u* = 0.34 (one modality missing), appropriately flagging reduced reliability to the clinical team (Table 17 and 18).

- **Missing Audio** (∼15% of cases): Accuracy drops 3.0% (87.3% → 84.3%).
- **Missing Physiological** (∼20% of cases): Accuracy drops 4.1% (87.3% → 83.2%).
- **Missing Video** (rare, < 2%): Accuracy drops 9.4% (87.3% → 77.9%).

**Table 17:** Out-of-distribution performance analysis.

OOD Scenario	Accuracy	Drop	Mean *u*	Mitigation Strategy
Gestational Age < 24w	74.2%	−13.1%	0.41	Collect more extreme preterm data
Post-surgical pain (new procedure)	78.9%	−8.4%	0.36	Fine-tune with 20–50 examples
MD-NPL dataset [40] (no fine-tuning)	79.8%	−7.5%	0.31	No fine-tuning; QWK = 0.761, AUC = 0.891
Home monitoring (non-NICU)	79.4%	−7.9%	0.38	Domain adaptation
COPE dataset (no fine-tuning)	81.4%	−5.9%	0.31	No fine-tuning; confirms generalization
Different ethnic population	82.1%	−5.2%	0.29	Cross-cultural validation ongoing

**Table 18:** Per-site accuracy for NICU-MM dataset.

Site	Country	Infants	Accuracy (%)	Meets >80% threshold
Hospital A	South Africa	156	87.3	Yes
Hospital B	Kenya	203	86.1	Yes
Hospital C	Nigeria	189	88.9	Yes
Hospital D	Ethiopia	142	82.9	Yes

#### 6.5.3 Out-of-distribution performance analysis.

**Per-site accuracy (NICU-MM):**

All four NICU-MM sites exceed the 80% clinical acceptability threshold.

#### 6.5.4 Deployment challenges and proposed solutions.

Based on pilot deployment at two clinical sites, practical challenges and solutions include: (1) real-time processing during high census addressed via staggered inference scheduling with priority for high-risk infants; (2) EHR integration addressed via FHIR-compliant API wrapper with OAuth 2.0 authentication; (3) clinician alert fatigue addressed via tunable thresholds and abstention for *u* > 0.5; (4) data drift addressed via monthly recalibration and federated updates; (5) privacy concerns for video/audio recording addressed via on-device processing, AES-256 encrypted storage, and 48-hour auto-deletion.

## 7. Conclusion

This work presents PANDIA, a novel neuro-symbolic framework for personalized infant pain assessment that successfully addresses key limitations of existing approaches while maintaining clinical practicality. Through the integration of concept bottleneck architectures, graph-based reasoning, meta-learning personalization, and federated training, PANDIA achieves performance that warrants further prospective validation (87.3% accuracy, 92.1% clinician acceptance) while maintaining computational efficiency suitable for edge deployment. Strong cross-dataset generalization and out-of-distribution robustness (79.8% on MD-NPL with no fine-tuning) are encouraging results; however, prospective clinical trials are required before deployment in live NICU settings.

Future research directions include expanding concept vocabularies, investigating cross-cultural generalization, developing real-time deployment systems with integrated clinical workflows, and extending the approach to other pediatric assessment challenges. We call for prospective randomized pilot studies in diverse NICUs and family medicine clinics to validate PANDIA’s impact on clinical outcomes and workflow efficiency.

## Supporting information

S1 Table Full annotation protocols for the iCOPE, NPAD, APN, and NICU-MM datasets. Covers annotation tools, frame sampling rates, episode segmentation criteria, annotator training, and consensus resolution procedures for all four datasets.
(PDF)

S2 Table Per-dataset performance breakdown for PANDIA and all baseline methods. Reports Accuracy (%), Quadratic Weighted Kappa (QWK), F1-Macro, and AUC for PANDIA and eight baseline methods across all four evaluation datasets (iCOPE, NPAD, APN, NICU-MM).
(PDF)

## Abbreviation:

AI: Artificial Intelligence
APN: Automated Pain in Neonates
AUC: Area Under the Receiver Operating Characteristic Curve
CNN: Convolutional Neural Network
COMFORT-B: COMFORT Behavior Scale
ECE: Expected Calibration Error
F1-Macro: Macro-averaged F1 Score
GDPR: General Data Protection Regulation
GNN: Graph Neural Network
HIPAA: Health Insurance Portability and Accountability Act
iCOPE: Infant COPE Dataset
MAML: Model-Agnostic Meta-Learning
MCE: Maximum Calibration Error
NICU: Neonatal Intensive Care Unit
NICU-MM: Neonatal Intensive Care Unit Multimodal Dataset
NIPS: Neonatal Infant Pain Scale
NPAD: Neonatal Pain Assessment Dataset
NPV: Negative Predictive Value
PANDIA: Personalized Adaptive Neuro-symbolic Data-fusion for Infant Assessment
PIPP-R: Premature Infant Pain Profile-Revised
PPV: Positive Predictive Value
QWK: Quadratic Weighted Kappa
SNR: Signal-to-Noise Ratio
TCN: Temporal Convolutional Network
ViT: Vision Transformer
