# Supplementary material for: PANDIA: Personalized neuro-symbolic multimodal fusion for interpretable neonatal pain assessment
Source: PLOS Digit Health. 2026 May 26;5(5):e0001442. doi: 10.1371/journal.pdig.0001442 (PMC13210372; doi:10.1371/journal.pdig.0001442)
Supplement: S1 Table — Covers annotation tools, frame sampling rates, episode segmentation criteria, annotator training, and consensus resolution procedures for all four datasets. (PDF) [file pdig.0001442.s001.pdf]

S1 Table: Annotation Protocols for All Four Datasets

S1 Table. Full Annotation Protocols for the iCOPE, NPAD, APN, and NICU-MM Datasets. Covers annotation tools, frame sampling rate, episode segmentation criteria, annotator training, and consensus resolution procedures. Full protocol documents and annotator training materials are available in the GitHub repository at `https://github.com/oussama123-ai/pandia/tree/main/supplementary`.

Parameter	iCOPE	NPAD	APN	NICU-MM	Notes / Rationale
**Annotation Tool**	ELAN v6.3 (MPI Nijmegen)	Custom web-based interface (USF)	ELAN v6.1 + MATLAB scripts	Label Studio v1.8.0 (open-source)	All tools export ELAN-compatible JSON; cross-tool reliability validated on 50 shared episodes.
**Modalities Annotated**	Video (facial AUs), Audio (cry)	Video (facial AUs), Physio (HR, SpO_2, RR)	Video, Audio, Physio (all)	Video, Audio, Physio (all)	Only modalities present at each site are annotated; missing modality flagged as `NA`.
**Frame Sampling Rate**	Every 5th frame (6 fps from 30 fps)	Every 3rd frame (10 fps from 30 fps)	Every 5th frame (6 fps from 30 fps)	Every 5th frame (6 fps from 30 fps)	Sampling rate chosen to balance annotation throughput with temporal resolution for AU detection.
**Episode Segmentation**	Procedural trigger (heel lance, venipuncture); 30 s pre + 60 s post-stimulus window	Pain event onset (±5 s); 10 s segments, 50% overlap	Stimulus onset (IM injection); 20 s pre + 60 s post window	Chart-documented event; 10 s segments, 50% overlap; min. 5 s duration	Boundaries capture full nociceptive arc (onset, peak, recovery).
**Pain Scale Used**	NIPS (0–7)	COMFORT-B (8–40) → 4-level ordinal	PIPP-R (0–21)	NIPS (primary); DAN (backup)	All scales harmonized: 0 = No pain, 1 = Mild, 2 = Moderate, 3 = Severe.
**Number of Annotators**	2 primary + 1 adjudicator	2 primary (NICU nurses, >5 yr) + 1 adjudicator	3 primary (round-robin) + 1 adjudicator	2 primary + 1 adjudicator (per site)	Adjudicator is always a board-certified neonatologist with ≥10 yr NICU experience.
**Annotator Training**	NIPS cert. (8 h); 50 calibration ep.; $\kappa \geq 0.70$ required	COMFORT-B (6 h); 30 cal. ep.; $\kappa \geq 0.65$ required	PIPP-R (6 h) + FACS AU (4 h); 40 cal. ep.; $\kappa \geq 0.70$ required	NIPS + DAN (10 h; EN/SW/YO/AM); 50 cal. ep.; $\kappa \geq 0.70$ required	Calibration episodes are held-out from model training and evaluation.
**Blinding Protocol**	Blinded to each other; no physio during video annotation	Blinded to each other during primary phase	Blinded to each other; AUs annotated before pain scale ratings	Blinded to each other and all other modalities during primary pass	Cross-modal blinding prevents physio/audio cues from contaminating facial AU annotations.
**Consensus Resolution**	Majority vote (2/3); adjudicator resolves ties	Discussion if $\lvert\Delta\rvert > 1$ on COMFORT-B; adjudicator decides	Majority vote; adjudicator reviews ≥1-level disagreements	Majority vote; >1-level discrepancies reviewed by both + adjudicator; reason code logged	Reason codes (fatigue, artifact, equivocal) logged for data quality audits.
**Inter-Rater Reliability**	$\kappa = 0.79$ (facial AUs)	$\kappa = 0.82$ (physiological)	$\kappa = 0.76$ (multimodal)	$\kappa = 0.78$ (facial C1–C4); $\kappa = 0.84$ (physio C9–C12)	All κ values indicate substantial agreement (Landis & Koch, 1977).
**QC / Audit**	10% re-annotation; >95% agreement required	5% audit; adjudicator review within 48 h	10% audit; AUs validated vs. published norms	10% per site (neonatologist, blinded); >95% achieved	QC audit episodes excluded from model training.
**Artifact / Rejection**	Motion blur >40% frames; mic clipping >3 s	Signal dropout >2 s; occlusion >50%	Dropout >2 s; cry <0.5 s	Dropout >2 s; illumination failure; >2-level disagreement (flagged, retained)	Flagged episodes retained with down-weighted loss ($w = 0.5$).
**Annotation Timeline**	Collected 2009–2012; re-annotated 2023	Collected 2017–2019; concepts added 2023	Collected 2014–2017; concepts added 2023	Prospective: Jan 2022 – Oct 2023	2023 re-annotations by annotators new to each dataset to avoid confirmation bias.

**Storage & De-id.**	HIPAA Safe Harbor; AES-256; NUH IRB #2007/412/B	HIPAA; AES-256; USF IRB #Pro00032180	HIPAA; AES-256; NTUH #201504033RINB	HIPAA+GDPR; AES-256; IRB-MRC-MHT-2022-001; KFU-REC-2023-10-28	All 18 HIPAA identifiers removed (45 CFR §164.514(b)(2)). IDs replaced with random UUIDs.

Abbreviations: AM = Amharic; AU = Action Unit (FACS); DAN = Douleur Aiguë du Nouveau-né; EN = English; ep. = episode(s); FACS = Facial Action Coding System; IM = intramuscular; NIPS = Neonatal Infant Pain Scale; PIPP-R = Premature Infant Pain Profile-Revised; QC = Quality Control; SW = Swahili; UUID = Universally Unique Identifier; YO = Yoruba. **Repository:** Full protocol documents, training slides, calibration sets, and Label Studio configs available at `https://github.com/oussama123-ai/pandia/tree/main/configs`.
