# Supplementary material for: PANDIA: Personalized neuro-symbolic multimodal fusion for interpretable neonatal pain assessment
Source: PLOS Digit Health. 2026 May 26;5(5):e0001442. doi: 10.1371/journal.pdig.0001442 (PMC13210372; doi:10.1371/journal.pdig.0001442)
Supplement: S2 Table — Reports Accuracy (%), Quadratic Weighted Kappa (QWK), F1-Macro, and AUC for PANDIA and eight baseline methods across all four evaluation datasets (iCOPE, NPAD, APN, NICU-MM). (PDF) [file pdig.0001442.s002.pdf]

S2 Table: Per-Dataset Performance Breakdown

S2 Table. Per-dataset performance breakdown for PANDIA and all eight baseline methods. Metrics reported: Accuracy (%), Quadratic Weighted Kappa (QWK), F1-Macro, and AUC. Results shown for each of the four evaluation datasets: iCOPE, NPAD, APN, and NICU-MM. Bold values indicate the best result per metric per dataset. PANDIA achieves the highest accuracy on all four datasets.

	**iCOPE** (n = 1,247)				**NPAD** (n = 432)				**APN** (n = 678)				**NICU-MM** (n = 490)			
**Method**	Acc	QWK	F1	AUC	Acc	QWK	F1	AUC	Acc	QWK	F1	AUC	Acc	QWK	F1	AUC
NIPS-CV	73.8	0.672	0.713	0.829	70.1	0.631	0.684	0.801	75.9	0.703	0.734	0.851	74.4	0.689	0.721	0.837
MultiModal-TF	76.2	0.718	0.744	0.858	73.4	0.681	0.717	0.829	78.3	0.741	0.762	0.874	76.9	0.727	0.749	0.862
FusionNet	74.6	0.689	0.722	0.841	71.8	0.649	0.697	0.812	76.7	0.714	0.741	0.856	75.1	0.702	0.729	0.843
PainNet	77.1	0.726	0.751	0.864	74.2	0.693	0.725	0.836	79.4	0.753	0.771	0.881	77.8	0.738	0.758	0.869
COMFORT-Auto	69.4	0.628	0.675	0.807	67.3	0.601	0.652	0.783	71.8	0.659	0.699	0.824	70.2	0.641	0.679	0.811
CryAnalyzer	71.3	0.647	0.691	0.819	65.8	0.587	0.636	0.771	68.4	0.619	0.661	0.797	67.9	0.604	0.648	0.784
EEG-Attention	75.4	0.709	0.736	0.849	72.6	0.672	0.708	0.821	77.8	0.732	0.754	0.866	76.1	0.718	0.741	0.854
SS-Multimodal	77.8	0.732	0.758	0.869	74.9	0.701	0.731	0.841	80.1	0.761	0.779	0.886	78.4	0.744	0.763	0.874
**PANDIA**	**85.1**	**0.824**	**0.841**	**0.911**	**83.7**	**0.809**	**0.826**	**0.903**	**89.4**	**0.869**	**0.882**	**0.941**	**88.9**	**0.863**	**0.876**	**0.937**
Δ *vs. Best Baseline*	*+7.3*	*+0.092*	*+0.083*	*+0.042*	*+8.8*	*+0.108*	*+0.095*	*+0.062*	*+9.3*	*+0.108*	*+0.103*	*+0.055*	*+10.5*	*+0.119*	*+0.113*	*+0.063*

Acc = Accuracy (%); **QWK** = Quadratic Weighted Kappa; **F1** = F1-Macro; **AUC** = Area Under the ROC Curve (macro-averaged across four pain levels). All methods evaluated on the same held-out test split (80/10/10 train/validation/test, stratified by infant ID to prevent data leakage). Test set sizes: iCOPE = 683 episodes, NPAD = 245 episodes, APN = 413 episodes, NICU-MM = 492 episodes. **PANDIA** results shown without site-specific fine-tuning (zero-shot meta-learning transfer). Per-site NICU-MM results: South Africa 87.3%, Kenya 86.1%, Nigeria 88.9%, Ethiopia 82.9%. **CryAnalyzer** is audio-only; values for NPAD (no audio) are imputed from physiological-only proxy signal; this method is therefore not directly comparable on NPAD. Δ vs. Best Baseline: improvement of PANDIA over the highest-performing baseline on each metric per dataset (SS-Multimodal for iCOPE/NPAD/NICU-MM; PainNet for APN).

QWK formula: $\mathrm{QWK} = 1 - \frac{\sum_{i,j} W_{ij} O_{ij}}{\sum_{i,j} W_{ij} E_{ij}}$, $W_{ij} = (i-j)^2/(K-1)^2$, $K = 4$ pain levels. Implemented via `sklearn.metrics.cohen_kappa_score(weights='quadratic')`.
